# In Silico Design and Characterization of a Rationally Engineered Cas12j2 Gene Editing System for the Treatment of HPV-Associated Cancers

**DOI:** 10.3390/ijms27021054

**Published:** 2026-01-21

**Authors:** Caleb Boren, Rahul Kumar, Lauren Gollahon

**Affiliations:** Department of Biological Sciences, Texas Tech University, 2500 Broadway, Lubbock, TX 79409, USA; caleb.boren@ttu.edu (C.B.); rahulkum@ttu.edu (R.K.)

**Keywords:** Cas12j2, CasΦ-2, CRISPR-Cas, HPV16/18, HPV-associated cancers, molecular dynamics simulations (AMBER), protein-RNA docking, gene therapy, off-target analysis, in-silico modeling

## Abstract

CRISPR-Cas9 systems have enabled unprecedented advances in genome engineering, particularly in developing treatments for human diseases, like cancer. Despite potential applications, limitations of Cas9 include its relatively large size and strict targeting requirements. Cas12j2, a variant ofCasΦ-2, shows promise for overcoming these limitations. However, its effectiveness in mammalian cells remains relatively unexplored. This study sought to develop an optimized CRISPR-Cas12j2 system for targeted knockout of the E6 oncogene in HPV-associated cancers. A combination of computational tools (ColabFold, CCTop, Cas-OFFinder, HADDOCK2.4, and Amber for Molecular Dynamics) was utilized to investigate the impact of engineered modifications on structural integrity and gRNA binding of Cas12j2 fusion constructs, in potential intracellular conditions. Cas12j2_F2, a Cas12j2 variant designed and evaluated in this study, behaves similarly to the wild-type Cas12j2 structure in terms of RMSD/RMSF profiles, compact Rg values, and minimal electrostatic perturbation. The computationally validated Cas12j2 variant was incorporated into a custom expression vector, co-expressing the engineered construct along with a dual gRNA for packaging into a viral vector for targeted knockout of HPV-associated cancers. This study provides a structural and computational foundation for the rational design of Cas12j2 fusion constructs with enhanced stability and functionality, supporting their potential application for precise genome editing in mammalian cells.

## 1. Introduction

Three different vaccines are currently approved in the United States for preventative use against HPV infection—Gardasil, Gardasil-9, and Cervarix. Upon completion of the vaccination series, individuals without prior HPV infection experience a nearly 100% reduction in their risk of developing HPV-associated cancers [1]. As the HPV infection progresses to the formation of pre-cancerous lesions, early medical intervention involves the local treatment with the removal and application of topical creams [2,3]. If left untreated, these pre-cancerous lesions can progress into invasive HPV-associated cancers with high metastatic potential [4]. In the advanced stages, the current standard of treatment involves the surgical resection of the primary tumor and a combination of interventions such as radiotherapy, chemotherapy, and immunotherapy [5].

Despite available vaccines and current treatment standards, HPV-associated cancers remain a significant global burden. Furthermore, studies have shown a shift from cervical cancer formation to tonsillar and rectal cancer incidence [6]. One study investigating trends in the global impact of HPV and the development of HPV-associated cancers found that in 2022 alone, there were over 1.5 million new diagnoses and >750,000 deaths [7]. Because the integration of E6/E7 drives carcinogenesis, various studies have investigated the therapeutic potential of CRISPR-Cas systems [8,9,10,11,12] in targeting them. A key molecular mechanism driving HPV-associated carcinogenesis is the integration of the viral genome into the host genome, leading to the sustained expression of the E6 and E7 oncogenes. These viral proteins promote malignant transformation by functionally inactivating the tumor suppressor proteins p53 and retinoblastoma (Rb), respectively, thereby disrupting cell-cycle regulation and apoptotic signaling. Because persistent E6 and E7 expression is required for both the initiation and maintenance of HPV-associated malignancies, these oncogenes have emerged as compelling therapeutic targets. Accordingly, multiple studies have explored the use of CRISPR–Cas systems [8,9,10,11,12] to disrupt E6 and E7 as a strategy to restore tumor suppressor function and inhibit cancer cell proliferation.

CRISPR-Cas systems have allowed researchers to make significant advancements in the areas of genomics, crop improvement, and the development of gene therapies [13,14]. These systems utilize CRISPR-derived RNA sequences that direct Cas endonucleases to complementary DNA targets, after which they are removed by cleavage [15]. The programmability of CRISPR-Cas systems is achieved through the design of a guide RNA (gRNA) that includes a CRISPR RNA (crRNA) segment, which facilitates the complexing of the gRNA with the Cas endonuclease, along with a protospacer sequence that directs the Cas:gRNA complex to a complementary DNA target [16]. While the CRISPR-Cas9 has quickly become the most widely used CRISPR-Cas gene editing system, it does have limitations. Its large size has made it challenging to encapsulate, and its strict protospacer adjacent motif (PAM) requirements make it difficult to identify targets within a given gene of interest at times [17]. As a result, research efforts have intensified to develop and engineer improved systems with more flexible properties [18,19,20].

In 2020, a novel member of the Class 2, Type V CRISPR/Cas system known as Cas12j2 (CasΦ-2) within the genome of the Biggiephage family of huge bacteriophages was identified [21]. Cas12j2 features a single, dual-function RuvC active site for crRNA processing and DNA cleavage, which contributes to its compact size. In terms of mass, Cas12j2 is approximately 75 kDa, making it half the size of both Cas9 and Cas12a [22,23]. Apart from its small size, it utilizes a minimally T-rich PAM sequence, allowing improved targeting capabilities for genes of interest [21]. When comparing Cas12j2 to the other nine subtypes (Cas12ja-n) of the Type V family, both similarities and differences exist with respect to structure, stability, and RNA binding [24]. In terms of cleavage activity, it is mediated by the aforementioned RuvC domain that is conserved across subtypes. Notable differences are related to gRNA architecture and protein-RNA interaction strategies. For example, subtypes such as Cas12i do not require a tracrRNA and can process their pre-crRNA, while others rely on crRNA-tracrRNA complexes [24]. Variations in RNA handling and domain organization suggest that determinants of structural stability and gRNA binding differ across subtypes. When considering this context, Cas12j2 represents a compact and comparatively less characterized Cas12 enzyme with the potential for a computational framework to guide future in vitro studies.

Overall, the combination of Cas12j2’s small size and targeting flexibility makes it an attractive candidate for development into a gene-editing system suitable for viral vector encapsulation and delivery [13,24]. Since its discovery, most studies investigating Cas12j2-based gene-editing systems have focused on plant models [25,26,27,28], with relatively limited work exploring its potential in mammalian cells [21,29]. At the time this study was conceptualized, Cas12j2 represented the foundational member of the Cas12j family available for investigation, as more recently reported variants such as Cas12j8 had not yet been described [30]. Subsequent developments demonstrating improved mammalian activity in related Cas12j variants build upon the foundational Cas12j2 architecture rather than diminishing its relevance. In this context, the present in silico analyses were designed to assess structural stability, guide RNA binding, and environmental sensitivity as factors relevant to the deployment of Cas12j2 in mammalian systems.

The goal of this study was to leverage computational modeling to design and characterize a CRISPR-Cas12j2 gene editing system for the knockout of a target gene of interest associated with HPV-induced cancers, thereby predicting its feasibility as a therapeutic approach to a globally devastating disease. In silico analysis enables the prediction and refinement of biomolecular interactions prior to experimental validation [18]. By leveraging the increasing accessibility and strength of established and emerging in silico tools, many of the interactions between the CRISPR-Cas gene editing system and a biological system can be computationally characterized [19]. While there are prophylactic vaccines available to prevent HPV-associated cancers, there are not currently any approved treatments that can fully eliminate the virus from infected cells [2]. This is primarily due to HPV’s ability to modulate the basal epithelial cells and maintain strict spatial and temporal control of its expression through its life cycle, effectively circumventing the host’s own immune defenses and medical interventions [2,25]. As part of their life cycle, high-risk strains can integrate into the host DNA or exist as episomes, allowing them to cycle between active and latent infections, which increases the challenges of effectively targeting them [26].

Our unique approach to the potential treatment of HPV-associated cancers involves incorporating the novel Cas12j2 endonuclease into a dual-gRNA gene editing system, which enables targeted gene knockout, whereas previous studies had only utilized a single-gRNA system. Additionally, the inclusion of markers such as the mCherry fluorophore allows for downstream validation in future in vitro studies. A plasmid co-expressing the novel Cas12j2 fusion construct and the dual gRNA system for target gene knockout was designed and developed to enable future functional validation and genome editing applications in vitro. This study provides a structural and computational foundation for the rational design of Cas12j2 variants with enhanced stability and functionality, supporting their potential application for precise genome editing in mammalian cells.

## 2. Results

### 2.1. Results for Structural Modeling of Cas12j2 Variants

The three Cas12j2 constructs analyzed in this study include the wild-type enzyme (WT), which serves as a structural baseline, and two engineered fusion constructs, Cas12j2_F1 and Cas12j2_F2. Cas12j2_F1 was previously developed by another research group [21] and contains C-terminal modifications. Cas12j2_F2 builds upon the existing Cas12j2_F1 design by including equivalent modifications to the N-terminal domain. The engineered Cas12j2_F2 fusion construct was designed to probe whether rational modifications alter global structural integrity or influence protein-gRNA interactions. Accordingly, structural differences observed among the variants are interpreted relative to WT behavior.

To investigate the impact of engineering Cas12j2 fusion constructs on the overall structure and function of the endonuclease prior to downstream in vitro studies, 3D structures of the Cas12j2 variants (WT, F1, and F2) were generated using ColabFold [27]. This was done to ensure that modifications did not significantly impact the overall structure in a way that interfered with its function and to generate models for downstream use. Results revealed that AlphaFold2 first shows the associated multiple sequence alignment (MSA). Additionally, the top five structural models were generated, each with an associated per-residue confidence (pLDDT) and predicted aligned error (PAE). This data provided a comprehensive overview of the structural integrity and reliability of the models.

**Figure 1:**
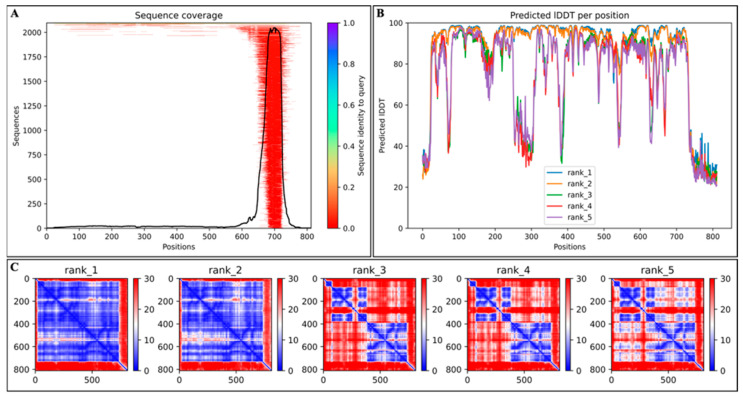
ColabFold Confidence Metrics for Cas12j2_F2. (**A**) The line graph shows the coverage plot for the multiple sequence alignment of Cas12j2_F2. (**B**) Line graph of per-residue predicted local distance difference test (pLDDT) scores for the five models predicted by AlphaFold2. (**C**) The predicted error alignment (PAE) for the five models predicted by AlphaFold2 is shown here.

Applying MSA in AlphaFold2 resulted in the predicted likelihood of correct structure and function of the E6 protein. The associated MSA of the variants utilized a collection of known proteins for homology and demonstrated high accuracy and consistency across the entire protein length (Figure 1A; Supplemental Figures S1A and S2A) [28]. In addition to the MSA, AlphaFold2 generated five models and provided both the per-residue confidence (pLDDT) (Figure 1B; Supplemental Figures S1B and S2B) and the predicted aligned error (PAE) (Figure 1C; Supplemental Figures S1C and S2C). For each variant model, the pLDDT was displayed as a line graph showing the per-residue confidence on a scale of 100. Most of the residues were scored above 70, indicating a high confidence in the structure integrity. Despite the relatively uniform pLDDT, there were some regions of low confidence in the predicted structure, particularly between positions 200–300 and after position 700, across the variants. The addition of the C-terminal tag is evident when comparing Figure 1C to Supplemental Figures S1B and S2B. Furthermore, the addition of the N-terminal can also be identified when comparing Figure 1B to Supplemental Figure S2B.

The PAE maps for each variant model provide an assessment of the relative positioning of residues across the model’s structure. This translates to high and low confidence for structure accuracy. The regions shaded blue show values with lower error, indicating high confidence. In contrast, the areas shaded red show values with higher error, indicating lower confidence. Across each variant, the top two models (rank_1 and rank_2) contained most of the lower error regions, showing high confidence in the overall folding (Figure 1C; Supplemental Figures S1C and S2C). The other three models (rank_3 through rank_5) showed the greatest number of high error regions, indicating low confidence in the overall folding. When comparing the PAE across all three variants, they all appear very similar to each other, indicating no major impact from the structural modifications. What is observed is a decrease in confidence around the terminal ends; however, the endonuclease core remains unchanged. Collectively, these results indicate that the engineered modifications do not disrupt the core structural integrity of Cas12j2, with the differences largely confined to the terminal regions, where flexibility is expected. This supports the conclusion that the overall folding and architecture of the WT, F1, and F2 variants are highly conserved.

**Figure 2:**
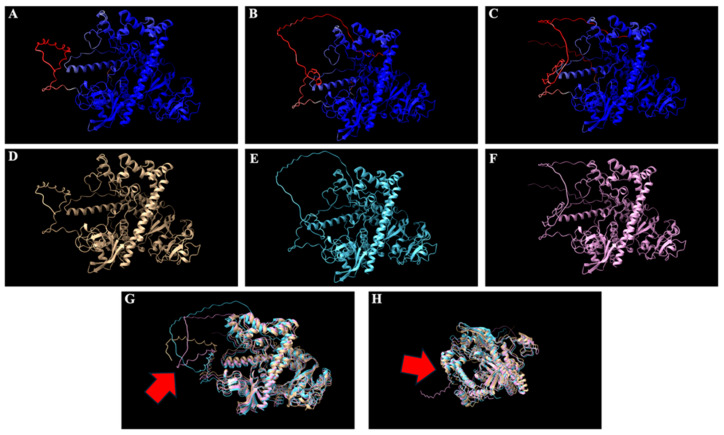
Structural Modeling of Cas12j2 Variants with ColabFold. (**A**–**C**) These ribbon models show the top-ranked models using B-factor mapping. Blue = high confidence and red = low confidence for (**A**) Cas12j2_WT, (**B**) Cas12j2_F1, and (**C**) Cas12j2_F2. (**D**–**F**) Top-ranked models colored to match the variant in overlay models: for (**D**) Cas12j2_WT, (**E**) Cas12j2_F1, and (**F**) Cas12j2_F2. (**G**,**H**) Comparison of the Cas12j2 variants using an overlay model to highlight differences, particularly at the (**G**) C-terminus and (**H**) N-terminus as indicated by red arrows.

Based on the high structural confidence, the top-ranked model for each of the Cas12j2 variants was selected for downstream analysis. Each of the models was overlaid with the others to highlight the differences at both the C-terminus (Figure 2G) and N-terminus (Figure 2H).

### 2.2. gRNA Candidate Design and Off-Target Analysis

**Figure 3:**
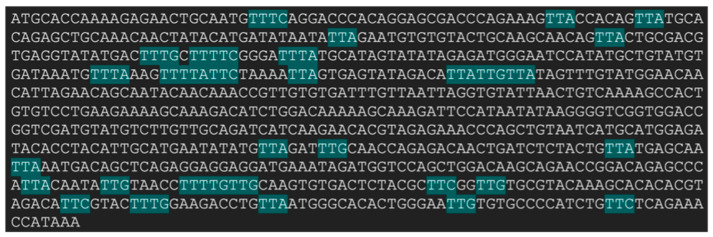
Identification of Cas12j2 Compatible PAM Sites within E6. This figure displays the nucleotide sequence of the HPV-16 E6 oncogene (GenBank–Accession # MH370217.1), annotated with blue highlights to identify potential PAM sites.

To effectively enable Cas12j2-mediated gene knockout of the HPV-16 E6 oncogene coding region inserted into mammalian cells, ensuring the quality of the gRNAs is a crucial aspect. The first consideration regarding system design was the target sequence to be removed, as discussed in the Section 4. Following the selection of the sequence, the next step involved annotating the 5′ and 3′ ends of the target sequence for suitable PAM sites that could potentially be used to design the gRNAs (Figure 3). Once identified, they were evaluated to determine if they were immediately upstream of a 20-nt sequence that could serve as a potential spacer (Table 1).

Once potential gRNAs targeting the 5′ and 3′ ends of the HPV-16 E6 oncogene were identified, their off-target editing potential needed to be evaluated [29,30,31,32]. Two software programs (CCTop (version 1.0) and CasOFFinder (version 1.0)) were selected to ensure robust analysis of candidate gRNAs’ off-target gene editing potential before further development [33].

To assess the off-target potential of candidate gRNAs, CCTop [34] was initially applied. The human reference genome GRCh37/hg19 was selected for predictive off-target analysis. Previous studies have shown that a large quantity of mismatches between the protospacer and target sequence can interfere with both recognition and downstream endonuclease activity [35]. Because of this, results were filtered to consider no more than four mismatches [29,30]. Once the initial parameters had been set within CCTop, each candidate gRNA was analyzed, and the results were compiled in a summary table (Table 2).

The data provided by the CCTop analysis of the candidate gRNAs revealed key information that, when considered together, narrowed selection for finalist rRNAs. CCTop output included general information, including the efficacy score and GC content percentage, which is provided, along with more specific details about off-target effects, such as location and quantity. The efficacy score is a CCTop-specific score that rates the likelihood of success of candidate gRNAs with a higher score. With GC content, the optimal range for gRNAs is considered to be between 40% and 70% [36,37]. For projecting potential off-target effects, predictions were categorized based on their genomic context—intergenic, intronic, and exonic. When analyzing the overall distribution of hits across the genomic context, the majority appeared to affect the intergenic and intronic regions, sparing the exonic regions. Because exonic off-targets are typically of greatest functional concern, this distribution suggests a reduced likelihood of protein-coding disruption. As such, intergenic and intronic hits were interpreted as being lower-priority off-targets in the context of this study.

For 5′ gRNA candidates, the efficacy score ranged from 0.52 to 0.70, and the GC content ranged from 45% to 70%. When evaluating the exonic off-target effects, a small range of 0–2 hits per gRNA was observed. gRNA4 had the lowest number of exonic hits (0), while gRNA1 had the most (2). The remaining gRNAs (2, 3, & 5) each had one exonic hit. After assessing the exonic hits, the next consideration was the total number of off-target events per gRNA. These results varied greatly, with gRNA3 exhibiting the most off-target predictions (3542) and gRNA5 showing the fewest off-target predictions (55). Taken together, the results predicted gRNA 1 and gRNA 5 to likely yield the most success.

The 3′ gRNA candidates’ efficacy scores ranged from 0.60 to 0.78, and the GC content ranged from 40% to 50%. Upon examining the exonic off-target effects, the range was similar to that of the 5′ gRNA candidates. gRNA3 had the least amount of hits (0), while gRNA5 had the most (2). The remaining gRNAs (1, 2, & 4) each had one exonic hit. Next, the total number of off-target events per gRNA was evaluated. The range was much smaller compared to the 5′ gRNA candidates. gRNA3 had the fewest off-target predictions (82), and gRNA2 had the most off-target predictions (328). Taken together, the predictive results indicated that gRNA 2 and gRNA 3 were the candidates most likely to yield success.

As previously mentioned, gRNA candidate finalists underwent an additional round of off-target analysis using a different software program to generate a more comprehensive analysis. The secondary software selected for evaluating off-target potential was CasOFFinder [34]. This software was selected due to its greater depth and accuracy in evaluation [38,39]. When preparing CasOFFinder for analysis, the reference genome (GRCh37/hg19) was used, and allowed a maximum of four mismatches. Once the initial parameters had been set within CasOFFinder, each of the gRNA candidates was analyzed. Results were compiled in the summary Table 3.

Unlike CCTop, CasOFFinder provides an additional layer of information by accounting for the impact of both DNA and RNA bulges on predicted off-target effects. In this context, a DNA bulge can be defined as a mismatch in the form of insertions or deletions within the genomic DNA relative to the spacer. RNA bulges have a similar definition; however, the mismatch is within the gRNA relative to the target DNA sequence. When considering the bulge range, the minimum considered was 1, and the maximum considered was 2. The mismatch range was allowed to vary and was determined by the predicted mismatches for each input gRNA sequence.

When analyzing the 5′-target gRNA finalists, candidates exhibit a remarkably similar overall DNA and RNA bulge profile. Starting with gRNA1, the DNA had a mismatch minimum of 2, resulting in 8 potential off-targets and a maximum of 4, yielding a total of 1482 potential off-targets. For the RNA mismatch of gRNA1, the minimum mismatch was 1, resulting in 2 potential off-targets and a maximum of 4, yielding a total of 6668 off-targets. Moving on to gRNA5, the DNA had a mismatch minimum of 2, resulting in 9 potential off-targets and a maximum of 4, yielding a total of 1083 off-targets. For the RNA mismatch of gRNA5, it had a mismatch minimum of 2, resulting in 10 potential off-targets, and a mismatch maximum of 4, resulting in a total of 13,511 off-targets. Although both candidates were comparable, gRNA5 was selected as one for inclusion in the Cas12j2 gene editing system.

The overall profiles of the two gRNA candidates are similar, yet distinct. When analyzing the DNA mismatch for gRNA2, it had a minimum of 1 mismatch, resulting in 4 off-target effects, and a maximum of 4 mismatches, yielding a total of 13,420 off-target effects. For RNA, gRNA2 had a minimum mismatch of 0, resulting in 2 off-target effects, and a maximum of 4 mismatches, which yielded 107,820, the highest value yet. In contrast, gRNA3 had an RNA minimum mismatch of 2, resulting in 3 off-target predictions and a maximum mismatch of 4360 off-target predictions. For the RNA, it had a minimum mismatch of 2, yielding 25 off-target predictions and a maximum of 4, yielding 19,830 off-target predictions. After comparing, it was determined that gRNA 3 was the best candidate and was selected for inclusion in the Cas12j2 gene editing system.

### 2.3. Structural Modeling of gRNAs

**Figure 4:**
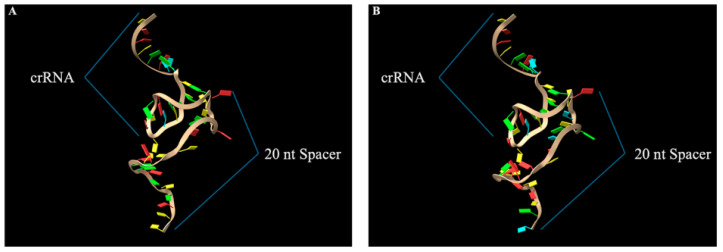
HPV-16 E6 Targeting gRNAs. (**A**) 3D model of the gRNA targeting the 5′ end of the E6 oncogene shown in ChimeraX with colors used to differentiate bases. (**B**) 3D model of the gRNA targeting the 3′ end of the E6 oncogene shown in ChimeraX with colors used to differentiate bases.

After careful design and evaluation of off-target potential, both the 5′ and 3′ targeting gRNAs were prepared for downstream protein-RNA docking simulations using RNA Composer (version 1.0) [40]. The software enabled the submission of gRNA sequences along with their secondary structure, provided in dot-bracket notation. This format was determined by visual inspection of experimentally validated figures described in the Cas12j2 discovery paper [21]. The resulting PDB files contained the predicted 3D structure for each gRNA and could then be opened using UCSF Chimera for visual inspection (Figure 4).

In both gRNA models, the upper region of the molecule represents the crRNA portion of the gRNA. The first nucleotide, immediately after the stem-loop structure, begins the 20-nucleotide protospacer. Despite differing sequences, a similar folding pattern is observed in both models. This supports confidence in the predicted structure and provides the final component needed prior to protein-RNA docking studies.

### 2.4. Protein-RNA Docking Analysis

While many factors contribute to the efficiency of gene editing systems for a given target, the stability of the Cas:gRNA interface is an important consideration. To investigate the impact of modifications to the Cas12j2 fusion constructs, the previously generated Cas12j2 variants and the gRNA structural models were docked utilizing HADDOCK 2.4. As the Cas12j2:gRNA binding interface has been experimentally validated, these docking simulations were run utilizing known interface residues, strengthening the overall validity.

For each of the 30 (3 variants × 2 gRNAs × 5 pH conditions) docking simulations, default settings for Protein-RNA docking were utilized. This configuration produced a total of 1000 rigid-body, 200 semi-flexible, and 200 solvent-refined models per simulation. No docking replicates were performed; each condition corresponds to a single HADDOCK 2.4 run. Models were clustered by RMSD, and clusters were ranked by the average HADDOCK score, with z-scores reported as a relative statistical descriptor of cluster scores within each run. The top cluster and its associated data were compiled into a table for further comparison (Table 4; Supplemental Table S1). The HADDOCK scores reported in Table 4 correspond to the average HADDOCK score of the top-ranked cluster for each docking condition, as determined using consistent clustering and scoring criteria across all simulations.

The primary metric that was used to evaluate the Cas12j2-gRNA complex was the HADDOCK score. This was because the HADDOCK score takes the following categories into consideration and then assigns an overall score, with lower HADDOCK score values indicating a more favorable overall docking outcome. As previously stated, Cas12j2_WT served as the baseline to which Cas12j2_F1 and Cas12j2_F2 were compared for relative binding favorability.

When considering the impact of pH, the Cas12j2-gRNA complexes were first individually assessed at each pH to determine pH-dependent trends. Docking favorability was evaluated using HADDOCK scores for the fixed protonation models. Beginning with Cas12j2_WT, when complexed with gRNA1, it showed the most favorable docking score at pH 5 and with gRNA2 at pH 6. For Cas12j2_F1, both gRNA1 and gRNA2 showed their most favorable docking scores at pH 6. With Cas12j2_F2, its most favorable docking score was at pH 6 for gRNA1 and at pH 5 for gRNA2. Based on this, it appears that the docking scores were most favorable between pH 5 and 6.

It is essential to note that the physiological cytosolic and nuclear pH in mammalian cells is maintained at near-neutral values (~7.4), and genome editing must ultimately occur under these conditions. As such, the enhanced stability observed at pH 5 and 6 is best interpreted as a measure of environmental sensitivity and structural robustness of Cas12j2–gRNA complexes, rather than as an indication of optimal in vivo performance. Mildly acidic conditions may be encountered transiently during intracellular trafficking or vesicular uptake, and stability under these conditions suggests that the engineered variants are not readily destabilized by pH fluctuations prior to nuclear localization.

After examining the pH at which Cas12j2 and its variants performed best, the next step was to compare the Cas12j2 variants against the wild-type structure to evaluate overall performance. Assessment of Cas12j2_WT showed an average HADDOCK score in the 30 s for both gRNA1 and gRNA2. When evaluating Cas12j2_F1, the average HADDOCK score is 108 for gRNA1 and 95.6 for gRNA2, representing a significantly higher score compared to the wild type. For Cas12j2_F2, the average HADDOCK score for gRNA1 was 68, and 64 for gRNA2. Taken together, it was determined that of the two variants, Cas12j2_F2 performed closer to the wild type than Cas12j2_F1.

### 2.5. Molecular Dynamics of Cas12j2-gRNA Complexes Using Amber

To investigate the overall behavior of the Cas12j2-gRNA complexes generated in the previous step, MD simulations were conducted over 200 ns. Systems were prepared using AmberTools, with ff14SB for the proteins and OL3 for the gRNAs in OPC water, which was set to 0.15 M NaCl in a 12 Å truncated octahedral box. After this, a 10-step minimization, heating, and equilibration protocol was conducted on the TTU HPCC (CPU: pmemd.MPI; GPU: pmemd.cuda). Simulations used an NPT ensemble set at 300 K, 1 bar (Langevin thermostat and Monte Carlo barostat), a 2 fs timestep with SHAKE on bonds to hydrogen, and PME electrostatics with a 10 Å real-space cutoff. Trajectories were analyzed for RMSD, RMSF, radius of gyration, and interface metrics (ΔBSA/SASA) to quantify complex stability across variants. Within this framework, docking results were interpreted as a proxy for initial complex formation/interface energetics under the fixed protonation models. The MD simulations were used to evaluate post-docking structural stability of the resulting complexes across pH conditions.

#### 2.5.1. Root Mean Square Deviation (RMSD) Analysis

**Figure 5:**
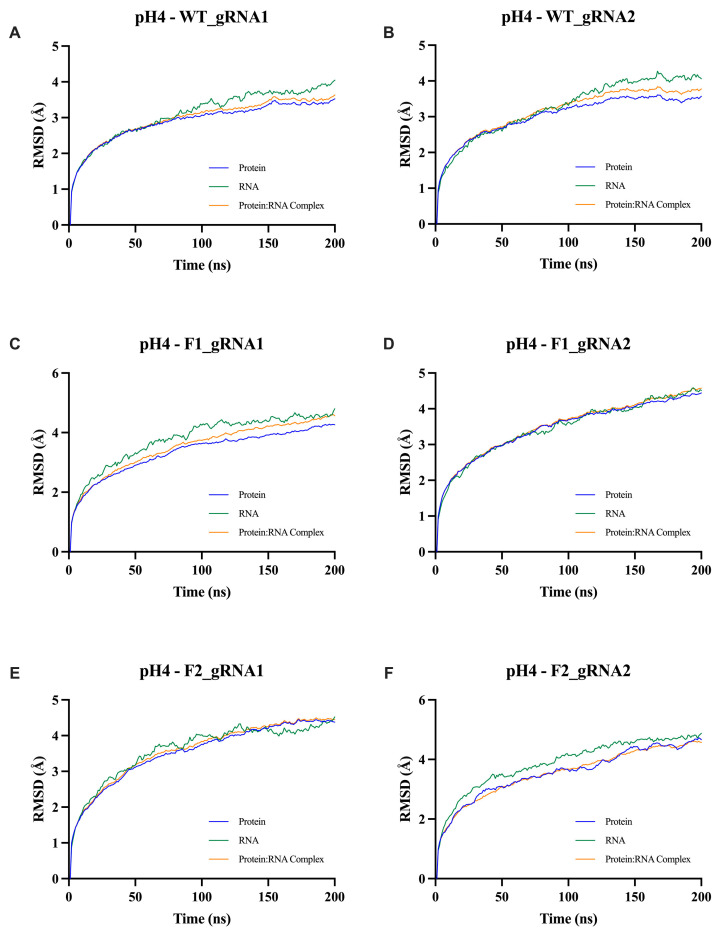
Root Mean Squared Deviation (RMSD) of Cas12j2-gRNA Complexes at pH 4. (**A**) WT_gRNA1 RMSD at pH 4. (**B**) WT_gRNA2 RMSD at pH 4. (**C**) F1_gRNA1 RMSD at pH 4. (**D**) F1_gRNA2 RMSD at pH 4. (**E**) F2_gRNA1 RMSD at pH 4. (**F**) F2_gRNA2 RMSD at pH 4.

To assess the overall structural stability of the Cas12j2–gRNA complexes under potential intracellular conditions, the first metric examined was the root-mean-square deviation (RMSD). This parameter provides a global measure of conformational changes over time relative to the initial structure and is commonly used to evaluate equilibration behavior and large-scale stability during molecular dynamics simulations. RMSD was calculated separately for the protein, the gRNA, and the combined protein–RNA complex to distinguish intrinsic RNA flexibility from structural changes occurring within the protein scaffold or the assembled complex (Figure 5; Supplemental Figures S3–S6).

Based on the data, it was determined that all Cas12j2-gRNA systems remained structurally stable throughout the 200 ns simulations across a pH range of 4–8. Convergence of the protein backbones occurred quickly within the first ~40 ns of the simulations. For Cas12j2_WT (Figure 5; Supplemental Figures S3–S6A,B), protein backbones stabilized between 2.5–3.5 Å, while the Cas12j2_F1 (Figure 5; Supplemental Figures S3–S6C,D) and Cas12j2_F2 (Figure 5; Supplemental Figures S3–S6E,F) fusion constructs stabilized between 3.0–4.0 Å. When considering the RNA, it consistently displayed a higher RMSD value (3.0–5.0 Å) related to its intrinsic flexibility. When examining the protein-gRNA complex RMSD, it fell between the trends of the individual protein and gRNA, indicating a stable intermolecular association.

#### 2.5.2. Root Mean Square Fluctuation (RMSF) Analysis

**Figure 6:**
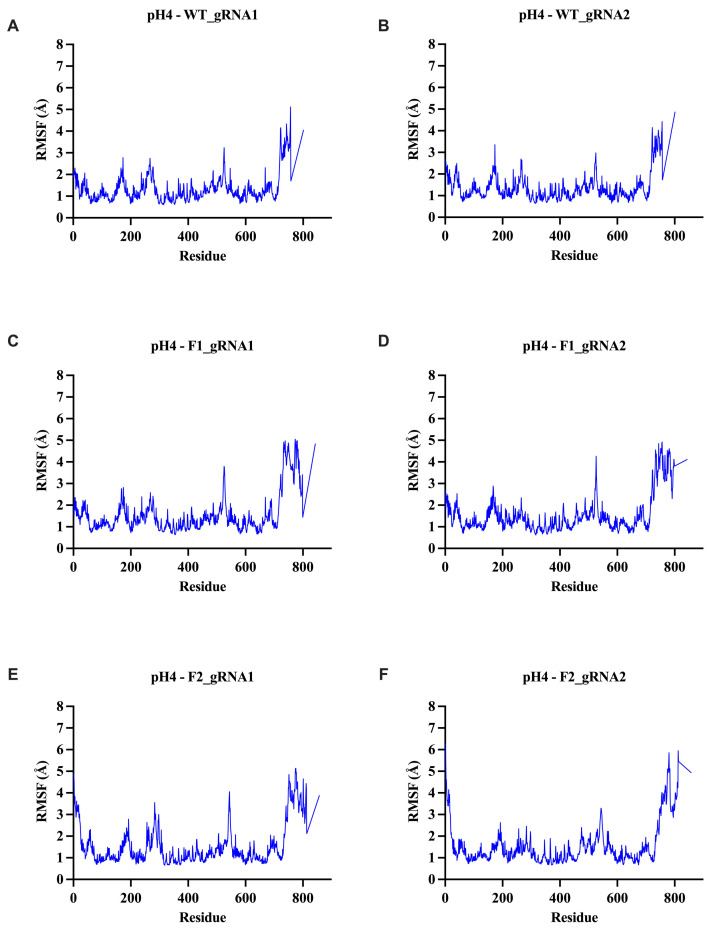
Root Mean Squared Fluctuation (RMSF) of Cas12j2-gRNA Complexes at pH 4. (**A**) WT_gRNA1 RMSF at pH 4. (**B**) WT_gRNA2 RMSF at pH 4. (**C**) F1_gRNA1 RMSF at pH 4. (**D**) F1_gRNA2 RMSF at pH 4. (**E**) F2_gRNA1 RMSF at pH 4. (**F**) F2_gRNA2 RMSF at pH 4.

To assess the local residue dynamics within the Cas12j2 variants, the root-mean-square fluctuation (RMSF) profiles of each construct were analyzed. RMSF provides insight into the positional variability of each residue relative to its average structure throughout the trajectory, allowing for the identification of flexible loops, structured domains, and other dynamic regions within the variants. Unlike RMSD, which reflects global conformational motion, RMSF captures localized fluctuations and therefore highlights which areas of the protein remain rigid or become more mobile under different simulation conditions. These RMSF profiles provided a detailed view of residue-level mobility across all variants and conditions (Figure 6; Supplemental Figures S7–S10).

Across all systems, the majority of the Cas12j2 variants displayed low to moderate fluctuations (~1–2 Å), which is consistent with a well-packed and structurally stable core. The peaks in RMSF can be explained by surface-exposed loops and regions containing peripheral elements. The catalytic core remained tightly constrained, while all constructs displayed higher fluctuations at the C-terminal tail, reflecting the intrinsic disorder in these regions rather than the instability of the structured domains.

#### 2.5.3. Radius of Gyration (Rg) Analysis

**Figure 7:**
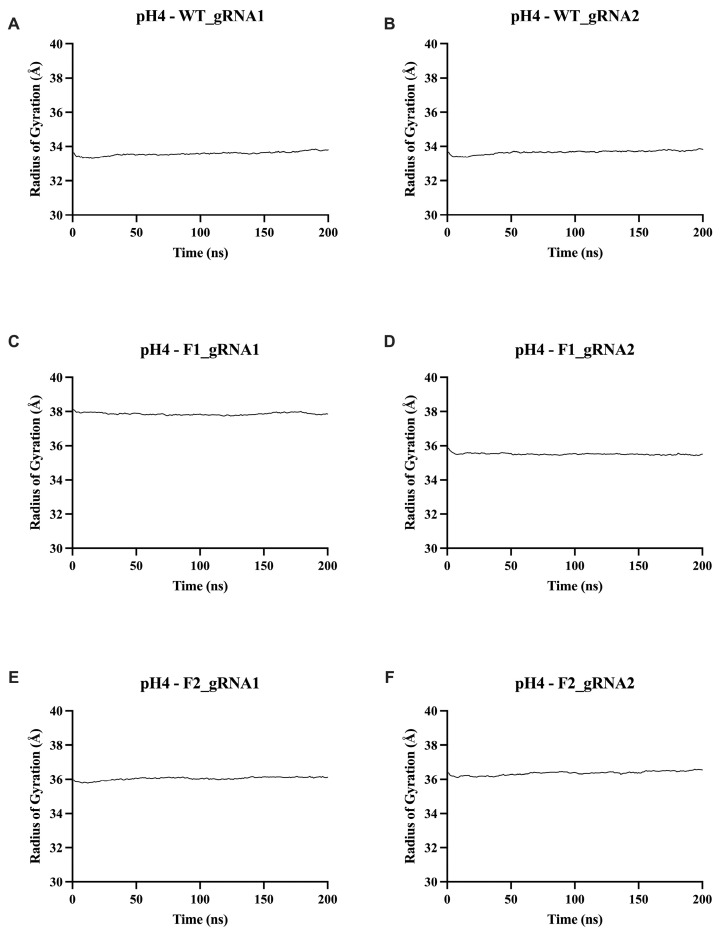
Radius of Gyration (Rg) of Protein-RNA Complexes at pH 4. (**A**) WT_gRNA1 Rg at pH 4. (**B**) WT_gRNA2 Rg at pH 4. (**C**) F1_gRNA1 Rg at pH 4. (**D**) F1_gRNA2 Rg at pH 4. (**E**) F2_gRNA1 Rg at pH 4. (**F**) F2_gRNA2 Rg at pH 4.

The radius of gyration (Rg) metric enables an assessment of a system’s overall compactness and equilibrium by comparing the average distance of its atoms from the center of mass. Smaller Rg values indicate a more compact structure, meaning greater stability, while larger Rg values indicate a less compact structure, allowing more flexibility. Across all conditions, the Rg traces were highly stable, showing only low-amplitude fluctuations around their respective mean values, showing no evidence of expansion or collapse (Figure 7; Supplemental Figures S11–S14).

When analyzing the wild-type Cas12j2 complexes, gRNA1 had a range of 33.59–33.96 Å, and gRNA2 had a range of 33.67–33.93 Å across all pH values, indicating that they are relatively stable. The engineered fusion constructs exhibited slightly higher mean Rg values (F1: gRNA1, 35.57–38.01 Å and gRNA2, 35.32–35.71 Å; F2: gRNA1, 35.55–36.44 and gRNA2, 36.36–36.75) due to their added mass; however, their time-dependent profiles were equally stable. As can be seen within the Cas12j2_F1 protein-gRNA complexes, there was a difference in Rg range between gRNA1 (Figure 7C and Supplemental Figure S11C) and gRNA2 (Figure 7C and Supplemental Figure S11C) at pH 4 and 5. This phenomenon was not observed in either the Cas12j2_WT or Cas12j2_F2 protein-gRNA complexes.

#### 2.5.4. Electrostatic Profile Analysis

**Figure 8:**
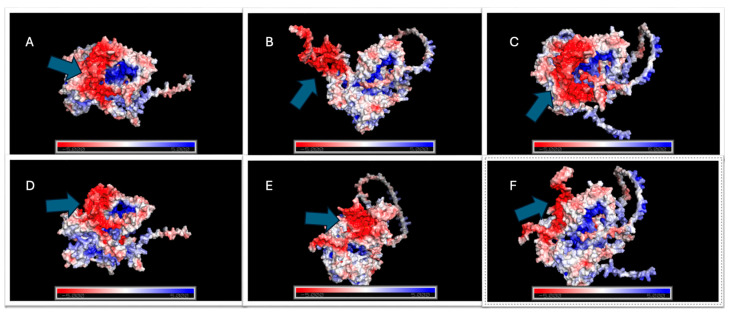
Electrostatic Surface Maps of Cas12j2 Fusion Constructs Bound to gRNAs at pH 4. (**A**–**C**) Cas12j2 fusion constructs bound to gRNA1. (**A**) Visualized electrostatic map of the complex Cas12j2_WT-gRNA1. (**B**) Visualized electrostatic map of the complex Cas12j2_F1-gRNA1. (**C**) Visualized electrostatic map of the complex Cas12j2_F2-gRNA1. (**D**–**F**) Cas12j2 fusion constructs bound to gRNA2. (**D**) Visualized electrostatic map of the complex Cas12j2_WT-gRNA2. (**E**) Visualized electrostatic map of the complex Cas12j2_F1-gRNA2. (**F**) Visualized electrostatic map of the complex Cas12j2_F2-gRNA2. When considering the figure legends, Red = negative potential, White = neutral potential, and Blue = positive potential. The arrows are used to indicate the Cas12j2-gRNA binding interface.

**Figure 9:**
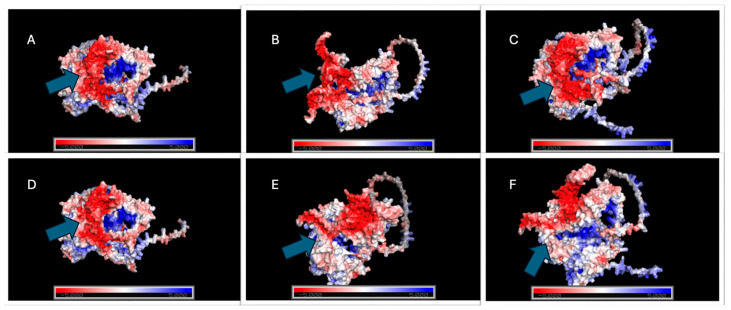
Electrostatic Surface Maps of Cas12j2 Fusion Constructs Bound to gRNAs at pH 8. (**A**–**C**) Cas12j2 fusion constructs bound to gRNA1. (**A**) Visualized electrostatic map of the complex Cas12j2_WT-gRNA1. (**B**) Visualized electrostatic map of the complex Cas12j2_F1-gRNA1. (**C**) Visualized electrostatic map of the complex Cas12j2_F2-gRNA1. (**D**–**F**) Cas12j2 fusion constructs bound to gRNA2. (**D**) Visualized electrostatic map of the complex Cas12j2_WT-gRNA2. (**E**) Visualized electrostatic map of the complex Cas12j2_F1-gRNA2. (**F**) Visualized electrostatic map of the complex Cas12j2_F2-gRNA2. When considering the figure legends, Red = negative potential, White = neutral potential, and Blue = positive potential. The arrows are used to indicate the Cas12j2-gRNA binding interface.

All structural models used for electrostatic analysis were obtained from the top-ranked docked complexes generated by HADDOCK2.4, using the lowest-energy cluster representatives for each Cas12j2–gRNA assembly. These structures were subsequently prepared and visualized in PyMOL (version 3.1.6.1), where electrostatic potentials were calculated through the APBS plugin following standard charge and radius assignments (Figure 8 and Figure 9; Supplemental Figures S15–S17).

Electrostatic analysis is commonly used because it provides insight into charge distribution, protein–RNA interaction stability, and pH-dependent behavior, helping to predict regions of favorable binding and conformational sensitivity that are not apparent from static structural models alone. With complexes containing gRNA1, no detectable shift in electrostatic potential across the examined pH range was observed in Cas12j2_WT. The surface polarity pattern at pH 4 remained indistinguishable from that observed at pH 8, indicating a highly stable charge environment throughout the RNA-binding interface. No changes in surface patch polarity or in the organization surrounding the binding channel were apparent under any condition.

In contrast, Cas12j2_F1 exhibited substantial pH-dependent variation. The electrostatic map at pH 4 differed considerably from that at pH 8, with broad charge redistribution occurring along the RNA-interaction surface. There was a marked reorganization of gRNA1 conformation, reflecting an increased sensitivity of the Cas12j2_F1–gRNA assembly to protonation-driven effects. The magnitude and spatial extent of these changes distinguished Cas12j2_F1 as the only gRNA1-associated variant that did not maintain electrostatic stability across pH conditions.

The Cas12j2_F2 demonstrated behavior that more closely aligned with that of Cas12j2_WT. The electrostatic landscape remained consistent between pH 4 and pH 8, with no meaningful alteration in either global charge distribution or local surface polarity. The gRNA1 conformation also remained unchanged, indicating that Cas12j2_F2, like Cas12j2_WT, sustains a stable protein–gRNA interface independent of pH.

For the same HADDOCK2.4-derived models complexed with gRNA2, the same PyMOL-APBS workflow was applied. Under the same conditions, Cas12j2_WT again showed complete stability across the full pH interval. No shifts in electrostatic polarity or redistribution of charged regions were observed between acidic and near-neutral conditions, demonstrating that Cas12j2_WT maintains a rigid electrostatic profile regardless of the bound gRNA.

Cas12j2_F1 displayed a similar trend. Unlike its interaction with gRNA1, Cas12j2_F1 exhibited no substantial electrostatic alterations when associated with gRNA2. The overall charge distribution and RNA-adjacent surface architecture were maintained from pH 4 to pH 8, suggesting that gRNA2 imposes structural constraints that suppress the pH-responsive features observed in the Cas12j2_F1-gRNA1 complex.

Cas12j2_F2 showed modest but measurable pH-dependent differences. Slight alterations in electrostatic potential were evident at the entrance of the RNA-binding cleft and along adjacent surface loops, accompanied by subtle adjustments in the conformation of gRNA2. While these effects were limited in magnitude and did not reflect a major rearrangement of the global electrostatic landscape, they were notably greater than any changes detected in Cas12j2_WT or Cas12j_F1 under equivalent conditions.

#### 2.5.5. Buried Surface Area

**Figure 10:**
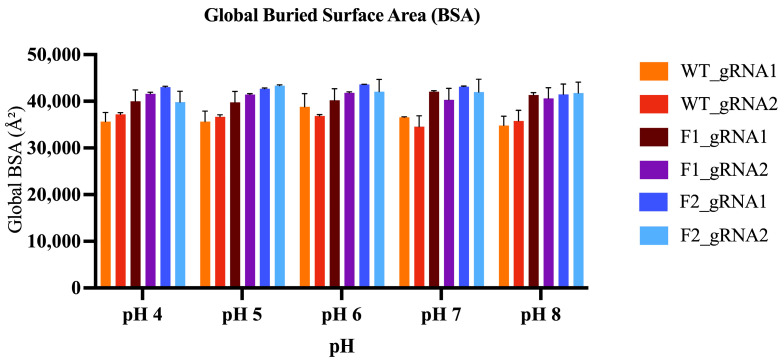
Global Buried Surface Area of Cas12j2-gRNA Complexes. This figure shows the mean BSA of each Cas12j2-gRNA complex, grouped by pH condition.

The buried surface area (BSA) metric allows for the assessment of a system’s interface size and degree of interface burial. When interpreting BSA, larger values indicate a greater extent of protein–RNA surface burial (i.e., a more extensive interface), whereas smaller values indicate reduced interface burial, which may reflect a more solvent-exposed or less engaged interface. To facilitate easy comparison, the mean BSA was present for each Cas12j2-gRNA complex, grouped by pH (Figure 10). Across pH 4–8, all Cas12j2–gRNA complexes maintained substantial interface burial, with BSA values remaining consistently high across conditions and showing little impact from pH. Overall, the fusion constructs exhibited comparable or higher BSA compared to the wild type, with F2 generally outperforming F1.

#### 2.5.6. Hydrogen-Bond Occupancy Analysis

**Figure 11:**
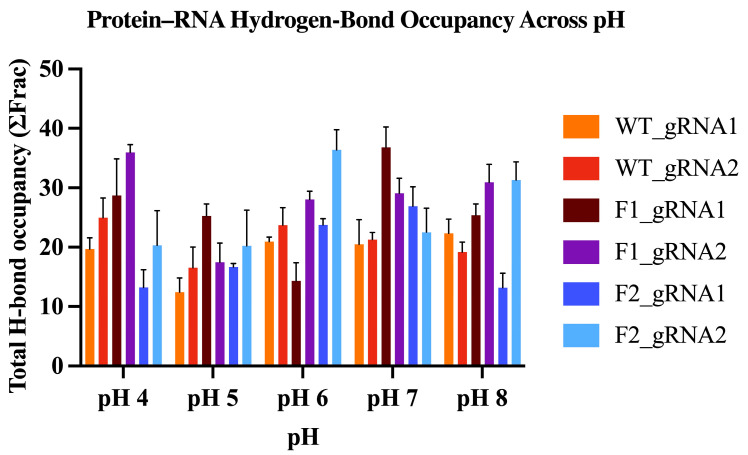
Hydrogen -Bond Occupancy of Cas12j2-gRNA complexes across pH. This figure shows the hydrogen-bond occupancy for the protein-gRNA complexes, grouped by pH condition.

The protein–RNA hydrogen-bond (H-bond) occupancy metric provides a complementary measure of interface contact persistence over time. When interpreting H-bond occupancy, higher values indicate a more persistent and/or denser hydrogen-bond network between Cas12j2 and the gRNA, consistent with a more stably engaged contact interface. In contrast, lower values indicate fewer and/or less persistent hydrogen bonds at the interface. To facilitate comparison across conditions, total interfacial H-bond occupancy was summarized as a cumulative occupancy metric (ΣFrac) for each Cas12j2–gRNA complex and grouped by pH (Figure 11).

Cumulative interfacial protein–gRNA hydrogen-bond occupancy (ΣFrac) varied as a function of both pH and gRNA identity. WT complexes were comparatively stable across pH, maintaining moderate ΣFrac values with a noticeable decrease at pH 5. In contrast, the engineered variants exhibited larger pH- and gRNA-dependent shifts in ΣFrac. Specifically, F1 showed elevated ΣFrac at pH 4 with gRNA2 and at pH 7 with gRNA1, whereas F2 reached its highest ΣFrac with gRNA2 at pH 6 and again at pH 8.

## 3. Discussion

### 3.1. Rational Design of a Dual gRNA Cas12j2 Gene Editing System

The rationale for designing this novel Cas12j2 fusion construct for future use with in vitro studies was to improve upon Cas12j2’s established efficiency in a single gRNA system. In the initial study documenting the discovery of Cas12j2, it was reported that its inclusion in a single gRNA editing system (Addgene: pPP441) had a 33% editing efficiency [21].

Cas12j2 possesses a C-terminal RuvC domain, which enables it to participate in both pre-crRNA processing and DNA cleavage. Due to the position of the C-terminal tag in the Cas12j2 single gRNA gene editing system, near the RuvC catalytic domain, it was thought that it could impact the catalytic activity, thereby reducing its efficiency [41]. Rather than simply swapping the C-terminal tag to the N-terminus, a tag of equivalent length was added to the N-terminus. The rationale was to design a Cas12j2 fusion construct that was comparable in size to the N- and C-terminal tags, would enhance its overall stability and functionality to the wild-type [42,43].

Within the cell, a critical trafficking pathway exists between the nucleus and cytoplasm. This network can be exploited by researchers by incorporating NLS sequences into their recombinant proteins. Upon recognition, the protein trafficking to the nucleus is enhanced [44]. NLS motifs have been found to contain stretches of positively charged amino acids. A variety of NLS motifs have been identified [44], each with its own unique architecture and mechanisms for interacting with the nuclear pore complexes (NPCs) of the nuclear envelope, facilitating entry into the nucleus. Such NLS motifs are commonly used in the design of recombinant proteins and include nucleoplasmin, simian virus (SV40) large T antigen, and c-myc [23]. One study evaluated the impact of SV40 quantity (0–4 copies) by comparing N-terminal, C-terminal, and dual-terminal configurations [45].

Based on results from these studies, two additional SV40 NLS sequences were selected for inclusion at the N-terminus. This resulted in the Cas12j2_F2 fusion construct containing four SV40 NLS sequences equally distributed to both termini. It was predicted that this would have a stabilizing effect on the overall Cas12j2 protein structure while preserving and potentially increasing the catalytic efficiency and enhancing nuclear accumulation.

Taken together, the Cas12j2_F1 and Cas12j2_F2 constructs were designed as stepwise fusion variants to evaluate how terminal tagging and NLS incorporation influence Cas12j2 structural stability, Cas12j2–gRNA interactions and predicted nuclear trafficking behavior. The Cas12j2_WT construct serves as a baseline reference, while Cas12j2_F1 and Cas12j2_F2 introduce incremental modifications intended to probe whether these design features alter enzyme behavior without disrupting the core protein architecture.

### 3.2. Structure Guided Engineering

The use of AlphaFold2-based modeling provided insight into the predicted structure and an assessment of the overall stability of Cas12j2 and its variants prior to future experimental validation [46]. Structural modeling using ColabFold revealed that the Cas12j2 wild-type (WT) and its fusion variants (F1 and F2) retained high overall structural confidence, as indicated by per-residue pLDDT scores.

The consistent confidence patterns among variants suggest that the addition of fusion tags did not noticeably disrupt the overall tertiary structure [46]. Such structural conservation is encouraging, as maintaining tertiary architecture is typically critical for Cas12-family nucleases to preserve catalytic efficiency and substrate recognition. However, localized decreases in pLDDT scores between residues 200–300 and beyond position 700 indicate regions of potential intrinsic disorder or low structural homology, which is consistent with previously reported flexible linker or tail regions in Class 2 CRISPR effector proteins [47]. These disordered regions may serve as hinge-like elements that facilitate conformational flexibility during target binding or R-loop formation, a feature often associated with efficient DNA cleavage mechanisms in other Class 2 nucleases [48]. PAE analysis further supported these findings, showing low predicted alignment error in core domains while revealing higher variability at terminal regions, particularly in constructs carrying N- or C-terminal extensions. This variability at termini may reflect flexibility rather than instability, potentially influencing accessibility for fusion tags or regulatory elements attached to these regions.

Collectively, the data indicate that the engineered modifications are structurally tolerated and maintain the general integrity of the Cas12j2 scaffold, while possibly introducing conformational flexibility that may have a positive effect on binding or catalytic activity. The accuracy of AlphaFold2 predictions is limited by the availability of homologous templates, and off-target analyses are limited by the completeness of the reference genome and algorithmic assumptions [49]. Future studies should therefore include experimental validation, such as cryo-electron microscopy, to confirm the folding of Cas12j2_F2. Such experimental follow-up would provide a crucial link between computational inference and biological function, confirming the translational relevance of these predictions.

### 3.3. Off-Target Predictions

As the use of CRISPR-Cas systems has become widespread, research efforts have been focused on increasing the overall efficiency and safety profile of gene editing systems [50,51]. A primary concern remains the potential off-target effects, particularly in the context of medical applications such as gene therapies [29,30,31]. Because experimental validation can be costly, predictive software has been developed to assess the off-target potential of gRNA candidates prior to experimental validation [32]. Since Cas12j2 is a relatively recent discovery, there is currently no software that explicitly evaluates the off-target potential of this system. Despite this, programs were identified that evaluated other Cas systems that shared the same 5′-TTN-3′ PAM site. The 5′ and 3′ gRNA candidates were assessed using both CCTop and Cas-OFFinder to evaluate their genome-wide off-target potential in the GRCh37/hg19 genome. This genome was chosen because it’s widely used in in silico modeling applications [52].

The CCTop software was used to initially screen the gRNA candidates, and the top two candidates for the 5′ and 3′ targets were then analyzed using the more extensive Cas-OFFinder [52,53]. The efficacy score, hit location, and total number of hits were all factors considered when narrowing the list of potential gRNA candidates. For the 5′ gRNA candidates, gRNA 1 and gRNA 5 had the lowest total number of hits and a low number of exonic hits. For the 3′ gRNA candidates, gRNA 1 and gRNA 3 had the lowest number of total hits and exonic hits; however, they had a relatively low efficacy score at 0.60. Both gRNA 4 and 5 were eliminated because they included an internal PAM site. Despite gRNA 2’s higher off-target potential based on the total number of hits, it was still selected because of its higher predicted efficacy score of 0.69 and its identical number of exonic hits.

As previously mentioned, the CasOFFinder software was used to screen the top two candidates that were determined following the CCTop analysis. This was due to CCTop only accounting for mismatches, while CasOFFinder considers DNA and RNA bulges. It has been discovered that mismatches of up to four nucleotides can be tolerated [54]. Because the software accounted for bulges within the alignment, this increased the overall total off-target hits collectively. When comparing the top two 5′ gRNA candidates, gRNA 5 outperformed gRNA 1. For the 3′ gRNA candidates, gRNA 3 outperformed gRNA 2.

Based on these results, it was determined that 5′ gRNA 5 and 3′ gRNA 3 were best supported by the data for inclusion within the Cas12j2 gene editing system, targeting the E6 oncogene for deletion. Despite the accuracy of off-target predictive software, limitations remain. A significant factor that these software do not take into consideration is chromatin and its known impact on off-target activity [33]. With the final gRNAs defined, they were 3D modeled and prepared for protein-RNA docking evaluation.

### 3.4. Docking and MD Stability of Cas12j2-gRNA Complexes

The decision to dock using experimentally validated interface residues strengthens the predictive value of our modeling. By constraining sampling to known contact regions, the risk of artifactual binding modes dominating the top clusters is reduced, a common issue in blind docking scenarios. Studies of protein–RNA docking emphasize that incorporation of prior interface data (or biochemical restraints) substantially improves the likelihood of retrieving near-native poses [55]. Thus, these docking outcomes (cluster selection, HADDOCK scores) have a higher chance of reflecting biologically realistic complexes than if molecular docking of the two molecules had been performed without any restraint.

The comparatively favorable HADDOCK scores for Cas12j2_F2 (closer to wild type) imply that its modifications preserve much of the native interface’s structural and energetic framework. Given that the scoring function of HADDOCK integrates van der Waals, electrostatic, desolvation, and restraint-violation energy terms, a near-wild-type score suggests that Cas12j2_F2 retains favorable inter-molecular contacts, electrostatic complementarity, and solvation properties [56]. In contrast, the substantially higher (less favorable) scores for Cas12j2_F1, particularly relative to wild type, imply that Cas12j2_F1’s mutations may impair one or more of these factors. Because protein–RNA interactions generally rely heavily on electrostatic complementarity, even modest disruption of charged residues or their spatial arrangement can reduce binding quality. The docking data suggest that Cas12j2_F1 may have degraded interface integrity, possibly due to poorer alignment, fewer favorable contacts, or altered solvation/desolvation energetics. Functionally, this could manifest as weaker binding affinity, reduced complex lifetime, or less efficient guide RNA recruitment, which are all factors detrimental to gene-editing activity. It is important to recognize the limitations inherent in computational docking.

Even though HADDOCK 2.4 is among the more prominent tools for protein–nucleic acid docking, and can incorporate flexibility and experimental restraints, the scoring functions are still approximations. For example, studies have shown that while docking can often sample reasonable binding modes, scoring functions may struggle to reliably distinguish among near-native and sub-optimal poses, especially for protein–RNA systems [57]. In particular, correct ranking by docking score (scoring success) does not always correlate with experimental binding affinity or functional activity [58]. Therefore, even though Cas12j2_F2 appears more favorable than Cas12j2_F1 by docking metrics, this does not guarantee that its binding affinity or functional performance will match WT in vitro or in cellular assays.

The RMSD, RMSF, and Rg profiles, taken together, provide a structural proxy for properties that influence Cas12j2 performance in gRNA binding and complex stability, while maintaining a catalytically competent scaffold. The rapid convergence of RMSD and stable Rg values suggest the global structure remains intact once the Cas12j2–gRNA complex is formed, consistent with preserving the conformational framework required for target engagement and R-loop formation. RMSF patterns further indicate that flexibility is largely confined to terminal and surface-exposed loop regions, whereas the structured core remains constrained, supporting preservation of the protein architecture. Variant-specific deviations are interpreted as increased conformational sensitivity, which may shorten complex lifetime or reduce the overall robustness of gRNA recruitment and retention. Importantly, these features map key barriers that have limited broader adoption of Cas12j2 in mammalian systems—intracellular stability and nuclear accumulation. These findings show that the intracellular stability of Cas12j2_F2 is favorable and that it is able to withstand extreme shifts in pH. In terms of nuclear accumulation, the presence of four SV40 NLS sequences will theoretically ensure optimal trafficking to the nucleus.

Moving beyond global stability to directly quantify interface behavior, we evaluated BSA and interfacial hydrogen-bond occupancy (ΣFrac) across the trajectories. BSA remained consistently high across pH 4–8, indicating that the protein–gRNA interface stays substantially buried under the tested conditions. In contrast, ΣFrac showed clearer pH- and gRNA-dependent shifts, suggesting that while the overall interface is preserved, the persistence of specific polar contacts at the interface is more sensitive to condition and gRNA identity. Together, these metrics provide quantitative support for preserved interface integrity in the engineered variants.

Genome editing in mammalian cells occurs under near-neutral pH conditions. Accordingly, the inclusion of mildly acidic conditions is interpreted as an environmental sensitivity analysis using fixed protonation states, rather than as an indication of optimal performance. Acidic compartments encountered during intracellular trafficking may impose transient protonation and electrostatic shifts that challenge protein–RNA interfaces. The observed maintenance of the global fold across conditions suggests that the engineered variants are not readily destabilized during intracellular transitions prior to nuclear localization.

### 3.5. Therapeutic Potential in HPV-Associated Cancers

The integration of HPV into the host genome drives carcinogenesis through the downstream expression of both the E6 and E7 oncogenes. This occurs due to the ability of E6 to promote the degradation of p53 using the ubiquitin-proteasome pathway [59,60]. It specifically accomplishes this by forming the E6/E6A/p53 complex, which involves the core domains of E6, ultimately suppressing p53 function. In the cell, p53 plays a crucial role as a transcription factor, regulating the cell cycle, apoptosis, and overall genomic stability [59]. The suppression of this key regulator creates ideal conditions for driving tumorigenesis.

The idea of using CRISPR-Cas9 systems to knock out HPV oncogenes is not novel, as previous studies have successfully demonstrated this ability [61,62,63,64]. Across these studies, a variety of delivery strategies have been employed to target the HPV E6 and E7 oncogenes, including plasmid-based CRISPR-Cas systems [61,62], AAV viral vectors [63], and nanoparticle-mediated delivery platforms [64]. It has been shown that disruption of these oncogenes can have effects such as restoration of p53 and Rb function [63], resulting in senescence [61] and apoptosis [63,64] as potential outcomes. Limitations such as the CRISPR-Cas system size have been a key obstacle identified by researchers [63].

What is unique about our approach is the specific selection of the Cas12j2 endonuclease, as there are limited studies that investigate its application to mammalian cells, especially in a dual-gRNA system. The size of most CRISPR-Cas systems poses a significant challenge when packaging them inside viral vectors. The use of this more compact system helps to overcome that obstacle, even with the additional modifications. In addition to its compact size, Cas12j2 offers a more flexible PAM site as previously discussed, allowing greater flexibility in targeting capabilities. Not only does its compact size and flexible PAM requirements make this a desirable system, but the intentional design of allowing both the Cas12j2 fusion construct within the same expression vector ensures the simultaneous delivery of Cas12j2 and the dual gRNA to each successfully transduced cell. Moreover, the inclusion of a mCherry fluorescent tag will allow for confirmation of successful package delivery in infected cells as well as more accurate identification of potential bystander effects.

### 3.6. Limitations and Future Directions

In this study, we present the rational design of a CRISPR-Cas12j2 gene editing system to enable gene knockout of E6, a primary oncogenic driver of HPV-associated cancers. This system is then analyzed computationally, utilizing evidence-based in-silico tools to evaluate the structural integrity of engineered Cas12j2 variants, the impact of intracellular pH on Cas:gRNA binding, and potential off-target effects. Despite this, there are limitations that should be considered, such as the accuracy of structural modeling and off-target analysis, and overall translation efficiency from in silico to in vitro and ultimately to in vivo. Despite its high-level accuracy (estimated to be 92.4%), there is still the possibility that the predicted structures used in this study were inaccurate, which would impact the reliability of downstream data [27]. The same concern for potential inaccuracies exists for both the docking phase and molecular dynamics simulations [65,66]. When it comes to off-target predictions, they rely on bulge and mismatch rules and do not consider other factors such as epigenetic states and chromatin [29]. Lastly, there can sometimes be a disconnect with the translation of in silico modeling to in vitro studies with regards to real-word functionality. This lack of translatability can occur due to simulations not accounting for (or able to simulate) the myriad unexpected cellular variables and immune responses [66]. Overall, this study presents a framework to rationally designing and computationally evaluating potential Cas12j2:gRNA complexes for use in a dual gRNA, CRISPR-Cas12j2 gene editing system. This study presents the results from successful in silico construction of an expression vector co-expressing both the Cas12j2_F2 fusion construct and a dual gRNA expression cassette to be applied in the next step—the in vitro construction and experimental validation of both efficiency and specificity of the in silico designed construct. If successful, this same framework could be applied to the E7 oncogene. Additionally, the structural and biophysical models can be refined to explore conformation robustness and probe stability.

## 4. Materials and Methods

### 4.1. Cas12j2 Sequence Retrieval and Variant Design

The wild-type Cas12j2 (Cas12j2_WT) nucleotide sequence utilized as the baseline for comparison of engineered Cas12j2 fusion constructs was retrieved from pPP441 (Plasmid #158801) through Addgene (https://www.addgene.org, accessed on 23 January 2025) [21]. The sequence file available from Addgene was imported and visualized in Benchling (https://www.benchling.com, accessed 23 January 2025). Within Benchling, sequences can be isolated and manipulated for designing fusion constructs that are suitable for downstream modeling. The first engineered Cas12j2 fusion construct (Cas12j2_F1) comes from the same expression plasmid pPP441 [21]. Pausch et al. fused two SV40 nuclear localization signals (NLS) and two FLAG tags to the C-terminus of Cas12j2, separated by standard linker sequences. This serves to improve nuclear import and provide a marker for downstream detection. The second engineered Cas12j2 fusion construct (Cas12j2_F2) kept the design of Cas12j2_F1 and fused two additional SV40 NLS sequences to the N-terminus. Following isolation (Cas12j2_WT and Cas12j2_F1) and design (Cas12j2_F2), Benchling was used to translate the DNA sequences into their amino acid (AA) code for downstream protein-RNA docking.

### 4.2. Design and Selection of Candidate gRNAs

Before generating potential gRNA candidates for Cas12j2-mediated gene knockout, the first step involved screening the gene of interest for potential PAM sites at the gene’s 5′ and 3′ ends [67]. The target gene selected for knockout was the HPV E6 oncogene, for which the reference sequence was obtained from GenBank (Accession #MH370217.1) [68]. Although Cas12j2 can recognize the 5′-TBN-3′ PAM site, it has been shown to have a higher affinity for the 5′-TTN-3′ PAM site variant, resulting in more efficient target recognition [21]. Following identification of PAM sites in the target gene, the 20 nucleotides immediately downstream of each PAM site were isolated for further consideration as potential protospacers, as this is the optimal length for Cas12j2 [21]. A total of ten protospacers were selected as potential gRNA candidates for initial screening—five for the 5′ end and five for the 3′ end of the target gene.

From here, candidate protospacers were filtered by constraints including acceptable GC content (40–80% range) and exclusion of an internal PAM (5′-TTN-3′) sites [36,69]. The next step involved running the candidate gRNAs through the CCTop software, where the candidates were prioritized based on their predicted efficacy score. The top two candidates for each target region were then subjected to further analysis using Cas-OFFinder. Final gRNA selection prioritized minimizing the predicted exonic off-target events first, followed by the overall predicted off-target burden. Intronic and intergenic hits were considered lower priority compared to exonic hits. Where CCTop efficacy and off-target metrics conflicted, the Cas-OFFinder profile was used to support the final selection. The use of two different software tools to assess the off-target potential was done as this is a key consideration in gRNA design [67].

### 4.3. Off-Target Analysis of Potential gRNA Candidates

Because Cas12j2 is a relatively new Cas variant, significantly fewer tools are available for assessing its off-target effects compared to more widespread Cas variants such as Cas9. Due to this, two web-based tools that screened for the 5′-TTN-3′ PAM site were selected—CC Top and Cas-OFFinder [34,70]. While not specific to Cas12j2, these tools are designed for Cas variants that utilize the particular PAM sequence. Of the two software, Cas-OFFinder can evaluate off-target prediction with more depth [29]. Utilizing both tools provided greater confidence in selecting minimally off-target gRNA protospacers that would effectively knock out the gene of interest.

Upon accessing CC Top, the initial approach involved pasting the E6 target sequence into the field for the query sequence. The 5′-TTN-3′ PAM site was selected from the PAM type dropdown menu, configuring the corresponding “Target Selection” and “Off-Target Prediction” fields. In the Target Selection field, the target site length was set to 20 nucleotides, and the two fields for the limitations on the target site for the 5′ and 3′ ends could be adjusted to filter the top 10 potential gRNA candidate sequences from the other potential gRNA protospacers. For the Section 3.3, the two key fields included the maximum total mismatches and the species selection. The maximum total mismatches allowed were set to be no greater than four nucleotides, and the organism selected for off-target analysis was human (*Homo sapiens GRCh37/hg19*).

Similar to CC Top, Cas-OFFinder features a user-friendly interface that enables easy modification of parameters, allowing for quick access to its off-target analysis. For consistency across off-target analysis software, the key parameter was kept unchanged. In this case, the PAM site 5′-TTN-3′ (FnCpf1 from *Francisella*) was chosen along with the model organism Human (*Homo Sapiens hg19*). The next step involved providing the query sequences field with the potential gRNA candidate 20-nucleotide sequences. Once completed, other variables, such as mismatch number, DNA bulge size, and RNA bulge size, are then taken into consideration. In this case, the following variables were selected: mismatch number (4), DNA bulge size (2), and RNA bulge size (2). At this point, the job was submitted, generating both a summary and a detailed table of the off-target analysis.

### 4.4. Approach to Structural Modeling of Cas12j2 Variants

The Cas12j2 variant theoretical structures were predicted utilizing the AlphaFold2_mmseqs2 pipeline accessed via ColabFold (v1.5.5) (https://github.com/sokrypton/ColabFold, accessed on 23 January 2025) [27]. In the default settings, only two parameters were altered. The first involved changing the num_relax field from zero to one with the goal of using energy minimization to resolve unrealistic geometries and improve the final structure for downstream applications [71]. The second parameter was modified by changing the template_mode from none to pdb100, allowing the software to access known models rather than rely on de novo prediction to increase confidence in accuracy [71,72]. After finalizing software settings, the Cas12j2 variants’ nucleotide sequences were converted to their amino acid sequences utilizing the translation tool in Benchling and entered into ColabFold for prediction. Once completed, 5 models were generated, and the top-ranked model was prepared for docking. Within UCSF Chimera (https://www.cgl.ucsf.edu/chimera/, accessed on 23 January 2025), the Dock Prep tool was utilized to make modifications, such as adding hydrogens, and assigning partial charges as needed [73,74,75,76]. The chain ID was then labeled “A”, and the newly prepared file was saved for protonation state modeling.

The 5′ and 3′ gRNA theoretical structures were modeled using the RNA Composer software [40]. The ribonucleotide sequences and secondary structure provided in dot-bracket from as determined from figures in Pausch et al. were entered into the software [21]. From this, the 3D models of the gRNAs were generated before being prepared for docking using UCSF Chimera, as described above for the Cas12j2 variants [75,76]. After preparation, the chain ID was set to “B”, and the file was ready for the next steps. To assess potential intracellular conditions for which the Cas12j2:gRNA complexes might encounter, protonated models of the three Cas12j2 fusion constructs were generated for pH values of 4, 5, 6, 7, and 8 using the pdb2pqr v3.6.2 software (https://ports.macports.org/port/pdb2pqr/, accessed on 3 February 2025) [77,78,79]. Following the generation of the protonated models, UCSF Chimera was utilized to convert files to the desired file type to ensure compatibility in downstream docking tools.

### 4.5. Protein-RNA Docking

To evaluate the interface binding activity of the Cas12j2 variants and gRNAs when exposed to potential intracellular conditions, protein-RNA molecular docking simulations were performed using the HADDOCK 2.4 software [80]. As previously described, the PDB files for the Cas12j2 variants, at selected intracellular pH concentrations, and gRNAs were prepared for docking using UCSF Chimera’s Dock Prep tool, and chain IDs were assigned. The pH-dependent effects were approximated by generating pH-specific protonation states for each structure prior to docking (static protonation models). Because protonation was held fixed during subsequent docking and molecular dynamic simulations, pH-related differences should be interpreted relative to the assigned protonation states.

Prior to initiating any simulations using HADDOCK 2.4, the first task was to identify the residues involved in the Cas12j2:gRNA binding interface from the work done by Pausch et al., in which they experimentally validated it using cryo-electron microscopy [21]. This is important because the residue information can be extracted to ensure that the in-silico models have the same active residues. This brings realism to protein-RNA docking because it guides the interaction to behave in the same manner as Cas12j2 has been experimentally shown to do. If the binding interface residues are unknown, the software will randomly dock the protein-RNA complex. To do this, the PDB file 7LYS was retrieved from RCSB PDB. This structure contains four different chains, but only chain A (Cas12j2) and chain B (gRNA) were retrieved [76,81]. The insights gained from these analyses were critical for defining the active residues in subsequent docking simulations, ensuring that the theoretical structures were grounded in experimentally validated interactions.

After inputting the necessary variant and gRNA combinations in HADDOCK 2.4 and including the active interface residues, the docking parameters were selected. The default recommended parameters for protein-RNA docking were utilized. This involved keeping the initial rigid-body structures at 1000, keeping the semi-flexible refinement models at 200, and keeping the final water-refined complexes at 200. At this point, the docking jobs were submitted and allowed to run anywhere from 12–24 h. The resulting structures were clustered and then ranked by a combination of factors, resulting in a HADDOCK score. The top-ranked structures were then selected for downstream analysis in molecular dynamics simulations to evaluate the impact of pH and variant modifications on binding activity.

### 4.6. Molecular Dynamic Simulations of Cas12j2-gRNA Complexes in Intracellular Conditions

Molecular dynamics (MD) simulations were employed to evaluate the structural stability and behavior of protein-RNA complexes generated by HADDOCK 2.4 in PDB format. For each of the pH conditions, MD simulations were initiated from structures prepared with pH-specific protonation states (static protonation models), and protonation states were held fixed over the course of the trajectories. The files were initially organized and prepared locally on a macOS system using Miniconda to utilize pdb4amber for standardizing residue naming and hydrogen placement [82,83]. Apart from the initial organization and cleaning, both the file preparation stage and the subsequent MD simulations were run on the Texas Tech University’s High Performance Computing Center (HPCC) CPU (Nocona and Quanah) and GPU (Matador) partitions. Both the CPU-parallelized pmend.MPI and GPU-accelerated pmend.cuda engines were required from the software Amber24 and AmberTools25. For the force fields, both ff14SB (Cas12j2) and OL3 (gRNA) were solvated in an OPC water model [81,84,85].

The tLeap program was used for both solvation and equilibration [81,83]. The protein-RNA complexes were centered in a truncated octahedral surrounded by a 12 Å buffer between the outside of the box [81,84]. The system was solvated with 0.15 M NaCl to simulate intracellular conditions [84,85]. Following solvation, a ten-step equilibration protocol was initiated to gradually relax solvent, ions, side chains, and backbone atoms while monitoring density stabilization [81]. In steps 1–5, the solvent and solute went through multiple rounds of restrained and unrestrained minimizations (1000–5000 cycles). In steps 6–9, short NPT molecular dynamics (5–15 ps each) allowed for relaxation of the backbone and side chains through the gradual decrease of positional restraints (from 5.0 to 0.0 kcal/mol·Å^2^). In step 10, an extended NPT equilibration (~1 ns) was performed to enhance stability prior to production of MD. The final topology (.prmtop) and coordinate (.inpcrd) files that were generated were used for downstream simulation steps. For each of the system and pH conditions, there were three independent MD replicates performed from the same equilibrated structure derived from the HADDOCK-generated complex. These replicates only differed in their initial atomic velocities assigned using distinct random seeds.

Once equilibrated, the final step involved performing production MD simulations using Amber24 with the pmend.cuda engine on the Matador partition of TTU HPCC to utilize GPU-acceleration [83]. The equilibrated systems underwent simulations in an NPT ensemble, where temperature was maintained at 300 K using the Langevin thermostat (γ = 1.0 ps^−1^) and pressure was maintained using the Berendensen barostat (1 bar) with a relaxation time of 2 ps [83,84,86,87]. When considering the timestep for the trajectories, a 2 fs timestep was used. The SHAKE algorithm was used to constrain all the bonds involving hydrogen atoms. The long-range electrostatics were treated with the Particle Mesh Ewald (PME) method, using a 10 Å cutoff [83].

### 4.7. Trajectory Analysis

Trajectory analyses were performed using the cpptraj module of AMBER24 to evaluate structural stability and conformational behavior of the simulated Cas12j2–gRNA complexes. All trajectories were first processed to remove imaging artifacts through periodic boundary condition correction, centering, and re-imaging. Only the equilibrated portion of the simulations (last 100 ns of the 200 ns production window) was used for analysis, and all reported values represent the mean across three independent replicates.

Backbone root-mean-square deviation (RMSD) was calculated for the protein, gRNA, and assembled protein–gRNA complex relative to the energy-minimized starting structure to assess global structural stability. Per-residue root-mean-square fluctuations (RMSF) were computed for protein Cα atoms to quantify local flexibility and identify dynamic regions within each variant. Global compactness was evaluated using radius of gyration (Rg) measurements across the equilibrated simulation window. These analyses were performed over the full 200 ns production trajectories, and reported values represent the average across three independent replicates for each system. To quantify protein–RNA interface packing during MD simulations, the buried surface area (BSA) was calculated from solvent accessible surface area (SASA) using $\mathrm{BSA}={\mathrm{SASA}}_{protein}+{\mathrm{SASA}}_{RNA}-{\mathrm{SASA}}_{complex}$ (Å^2^) [88]. In addition, interfacial hydrogen bonds between Cas12j2 and the gRNA were quantified in cpptraj (AmberTools). H-bonds were defined by standard geometry (distance ≤ 3.5 Å; angle ≥ 135°) and computed in both directions (protein → RNA and RNA → protein) [89]. H-bond occupancy was evaluated over the final 100 ns of each trajectory using a stride of 10 frames. Per-bond occupancies (fraction of analyzed frames) were summed across unique interfacial H-bonds (ΣFrac) and reported as mean ± SD across three MD replicates per condition.

### 4.8. Electrostatic Surface Mapping and Solvent Accessible Surface Area Analysis 

Following the identification of the top-ranked docking clusters from HADDOCK 2.4, electrostatic surface potential mapping was performed to qualitatively assess charge distribution at the protein–RNA binding interface. The PDB files corresponding to the best-scoring complexes were prepared and visualized in PyMOL (version 3.1.6.1) [90], where electrostatic potentials were calculated using the APBS plugin with standard charge and radius assignments. This approach provides a qualitative visualization of the electrostatic surface potential across the protein surface of interest. The maps generated allow for a comparison of charge between Cas12j2 variants and their associated gRNAs, providing insight into how electrostatic distribution may contribute to molecular recognition and stability under simulated intracellular conditions. While this method does not yield quantitative electrostatic values, the visualization of localized positive and negative patches allows for the identification of potential electrostatic hotspots that may contribute to the stabilization of protein–RNA interactions.

Solvent Accessible Surface Area calculations were carried out to quantify the extent of solvent exposure for residues within the docking interface at different pH values. The GETAREA server (https://curie.utmb.edu/getarea.html, accessed on 20 November 2025) [91] was utilized to carry out these calculations. The same docking-derived PDB structures that were used in PyMOL were uploaded to the GETAREA server. A water probe radius of 1.4 Å was specified to approximate the size of a solvent molecule [92]. No additional advanced options were changed, and default parameters were applied in order to maintain consistency across all samples. The resulting data from GETAREA provided total SASA values as well as per-residue contributions, which allow for direct comparison of solvent exposure between Cas12j2 variants at different pH values. These results, when integrated and interpreted in conjunction with docking scores and electrostatic surface potential maps, provide a comprehensive view of how pH-dependent conformational dynamics and variant-specific modifications may influence binding affinity and stability at the protein–RNA interface. 

### 4.9. Design and Production of Expression Vector

To facilitate the in vitro validation studies, the Cas12j2_F2 fusion construct and both gRNAs were designed to be co-expressed in a mammalian expression vector. The expression vector was designed based on the existing pPP441 expression vector [21]. The first synthetic fragment was designed to keep Cas12j2_F2 under the control of the CBA promoter for use in pPP441 and included the P2A sequence. After this sequence, the mCherry fluorophore was inserted to allow downstream visualization and validation. A secondary synthetic fragment was designed that included the dual gRNA expression cassette. Each gRNA is under the control of a U6 promoter and terminated by a Poly(T) sequence. A 50-nt non-coding sequence was included to separate the two-gRNA expression cassettes to optimize their expression. It was determined that these two synthetic fragments could be incorporated into the minimal pUC57 expression vector. When it came to production, this design was sent to Gene Universal (Newark, DE) for commercial production to ensure accuracy and quality of the design. The company utilized multiple quality control measures, including multiple stages of Sanger sequencing and restriction digest analysis, to ensure 100% accuracy in design.

## 5. Conclusions

In this study, the rational design of a CRISPR-Cas12j2 gene editing system is presented to enable gene knockout of the E6 oncogene as a potential therapeutic for HPV-associated cancers. This system was analyzed utilizing evidence-based in-silico tools to evaluate the structural integrity of engineered Cas12j2 fusion constructs, the impact of intracellular pH on Cas:gRNA binding, and potential off-target effects of gRNA candidates. This computational framework determined that the novel fusion construct did not significantly alter the structural integrity of Cas12j2 when compared to a known, experimentally validated Cas12j2 fusion construct. The predicted structure was also not impacted by potential intracellular pH conditions that could be encountered during transport to and expression in a target cell. The use of off-target analysis software facilitated the careful design of two high-quality gRNAs targeting the 5′ and 3′ ends of the E6 oncogene with low potential for off-target effects.

Limited studies have been conducted exploring the application of Cas12j2 systems in the gene editing of mammalian cells, particularly with respect to their therapeutic application in cancer [93]. This study serves as a computational framework to guide the design and evaluation of CRISPR-Cas systems, prior to in vitro validation. Not only will this approach save time and money [61], but it could also increase the success rate when transitioning to in vitro studies [94]. Based on this approach, an expression vector that co-expressed their novel Cas12j2 fusion construct and a dual gRNA expression cassette was designed. During the process, in silico analyses predicted that each successfully transduced target cell would receive both components of the gene editing system. The inclusion of a dual FLAG tag and mCherry fluorophore will facilitate efficacy in evaluating in vitro applications. Future studies will focus on the optimization of encapsulating this gene editing system within a novel viral vector and then experimentally validating its therapeutic potential for the treatment of HPV-associated cancers through Cas12j2-mediated knockout of the E6 oncogene.

## Figures and Tables

**Table 1:** Candidate gRNAs for the HPV E6 oncogene from GenBank (Accession #MH370217.1).

E6 gRNA Candidates						
Target Region	Features	gRNA 1	gRNA 2	gRNA 3	gRNA 4	gRNA 4
5′ Target	PAM	TTT	TTA	TTA	TTA	TTA
	20 nt sequence	CAGGACCCACAGGAGCGACC	CCACAGTTATGCACAGAGC	TGCACAGAGCTGCAAACAAC	GAATGTGTGTACTGCAAGCA	CTGCGACGTGAGGTATATGA
3′ Target	PAM	TTT	TTA	TTG	TTC	TTT
	20 nt sequence	GCAACCAGAGACAACTGATC	AATGACAGCTCAGAGGAGG	TGCGTACAAAGCACACACGT	GTACTTTGGAAGACCTGTTA	GGAAGACCTGTTAATGGGCA

**Table 2:** CCTop Analysis of Off-Target Gene Editing Potential for Candidate gRNAs in GRCh37/hg19.

E6 Region 5′-gRNAs Candidates					
Selection Parameters	gRNA 1	gRNA 2	gRNA 3	gRNA 4	gRNA 5
PAM Site	TTT	TTA	TTA	TTA	TTA
20-nt Target Sequence	CAGGACCCACAGGAGCGACC	CCACAGTTATGCACAGAGCT	TGCACAGAGCTGCAAACAAC	GAATGTGTGTACTGCAAGCA	CTGCGACGTGAGGTATATGA
Efficacy Score	0.66	0.57	0.52	0.58	0.70
G/C Content Percentage	70%	50%	50%	45%	50%
Intergenic Hits	7	7	9	11	8
Intronic Hits	11	12	10	9	11
Exonic Hits	2	1	1	0	1
Total Off-Target Hits	97	296	3542	235	55
E6 Region 3′-gRNAs Candidates					
Selection Parameters	gRNA 1	gRNA 2	gRNA 3	gRNA 4	gRNA 5
PAM Site	TTT	TTA	TTG	TTC	TTT
20-nt Target Sequence	GCAACCAGAGACAACTGATC	AATGACAGCTCAGAGGAGG	TGCGTACAAAGCACACACGT	GTACTTTGGAAGACCTGTTA	GGAAGACCTGTTAATGGGCA
Efficacy Score	0.60	0.69	0.63	0.78	0.65
G/C Content Percentage	50%	50%	50%	40%	50%
Intergenic Hits	8	7	10	9	7
Intronic Hits	11	12	10	10	11
Exonic Hits	1	1	0	1	2
Total Off-Target Hits	166	328	82	262	168

**Table 3:** Summary Table of CasOFFinder Analysis of Candidate gRNAs in GRCh37/hg19.

E6 Region 5′-gRNA Finalists					
gRNA	Bulge Type	Observed Summary (Min/Max)	Bulge Length (nt)	Mismatch Count (n)	Potential Off-Target Sites (n)
gRNA 1	DNA	Minimum	1	2	8
		Maximum	2	4	1482
	RNA	Minimum	1	1	2
		Maximum	2	4	16,668
gRNA 5	DNA	Minimum	1	2	9
		Maximum	2	4	1083
	RNA	Minimum	1	2	10
		Maximum	2	4	13,511
E6 Region 3′-gRNA Finalists					
gRNA	Bulge Type	Observed Summary (Min/Max)	Bulge Length (nt)	Mismatch Count (n)	Potential Off-Target Sites (n)
gRNA 2	DNA	Minimum	1	1	4
		Maximum	2	4	13,420
	RNA	Minimum	1	0	2
		Maximum	2	4	107,820
gRNA 3	DNA	Minimum	1	2	3
		Maximum	2	4	4360
	RNA	Minimum	1	2	24
		Maximum	2	4	19,830

Note 1: Cas-OFFinder analyses were performed using thresholds of ≤4 mismatches and ≤2-nt bulges for both DNA and RNA. Table 3 summarizes, for each gRNA, the minimum and maximum bulge lengths observed among returned off-target alignments and the corresponding mismatch counts, reported separately for DNA-bulge and RNA-bulge modes. “Potential Targets” indicates the number of genomic hits returned by Cas-OFFinder for the specified bulge length and mismatch count within these thresholds. Note 2: Table Header Definitions—(1) Bulge Type: indicates bulges were allowed in either DNA or RNA, (2) Observed Summary (Min/Max): indicates whether the row reports the minimum or maximum observed condition among returned hits., (3) Bulge Length (nt): number of inserted/deleted bases (0–2) in the reported alignment., (4) Mismatch Count (n): number of mismatched positions in the reported alignment (0–4)., and (5) Potential Off-Target Sites (n): number of genomic hits returned under the specified bulge length and mismatch count within the chosen thresholds.

**Table 4:** HADDOCK 2.4 Summary Table.

Variant ID	gRNA	pH	Cluster #	Cluster Size	HADDOCK Score (±SD)	RMSD (±SD)	vdW Energy (kcal/mol ± SD)	BSA (Å^2^ ± SD)
Cas12j2_WT	1	4	2	22	33.0 ± 16.6	16.3 ± 0.1	−85.0 ± 8.8	3093.2 ± 134.4
		5	4	6	30.1 ± 38.6	0.8 ± 0.5	−102.0 ± 13.9	3293.0 ± 188.1
		6	2	19	32.6 ± 16.2	16.3 ± 0.1	−81.2 ± 3.8	3035.6 ± 108.0
		7	2	17	43.6 ± 7.0	16.3 ± 0.1	−85.2 ± 8.7	3039.6 ± 114.8
		8	2	19	32.6 ± 16.2	16.3 ± 0.1	−81.2 ± 3.8	3035.6 ± 108.0
	2	4	2	16	46.3 ± 11.2	6.4 ± 0.3	−79.2 ± 11.3	2912.0 ± 249.9
		5	2	11	46.5 ± 11.4	6.5 ± 0.3	−80.2 ± 11.6	2935.2 ± 248.1
		6	2	17	22.4 ± 8.2	6.3 ± 0.1	−97.8 ± 16.7	3455.1 ± 306.4
		7	2	11	26.2 ± 9.2	6.3 ± 0.1	−92.9 ± 9.5	3223.8 ± 143.7
		8	2	17	32.1 ± 11.1	6.2 ± 0.0	−86.5 ± 3.4	3140.2 ± 131.9
Cas12j2_F1	1	4	2	11	108.1 ± 23.9	12.5 ± 0.6	−62.2 ± 14.2	2199.6 ± 230.7
		5	1	19	109.0 ± 11.1	13.0 ± 0.0	−64.7 ± 8.2	2168.7 ± 302.3
		6	3	6	99.6 ± 5.1	12.6 ± 0.3	−93.5 ± 9.1	2773.5 ± 235.9
		7	5	6	95.4 ± 35.7	10.9 ± 0.5	−73.4 ± 14.4	2638.0 ± 369.1
		8	8	4	132.4 ± 27.9	10.7 ± 0.2	−60.3 ± 3.9	2307.6 ± 135.7
	2	4	3	6	82.8 ± 22.2	14.0 ± 0.1	−89.5 ± 10.3	2876.9 ± 140.8
		5	5	6	96.2 ± 13.0	4.6 ± 0.1	−96.5 ± 4.5	3260.3 ± 263.8
		6	5	8	91.8 ± 14.8	8.6 ± 0.4	−80.4 ± 11.9	2647.2 ± 313.9
		7	5	6	103.8 ± 29.8	7.9 ± 0.1	−70.2 ± 18.6	2614.7 ± 314.2
		8	3	7	103.6 ± 20.3	12.8 ± 0.1	−85.7 ± 8.8	2680.9 ± 204.7
Cas12j2_F2	1	4	2	12	40.7 ± 11.5	17.1 ± 0.3	−94.5 ± 3.2	3079.6 ± 262.1
		5	1	15	109.4 ± 12.4	11.5 ± 0.4	−83.3 ± 6.8	2632.5 ± 258.8
		6	2	7	51.4 ± 5.0	13.6 ± 0.2	−90.7 ± 2.6	2890.7 ± 102.7
		7	3	5	70.7 ± 19.7	12.9 ± 0.7	−80.4 ± 18.4	2854.2 ± 243.1
		8	3	8	66.6 ± 15.3	16.1 ± 0.1	−86.1 ± 5.9	2935.4 ± 235.7
	2	4	5	4	79.5 ± 21.0	12.6 ± 0.1	−77.2 ± 9.8	2695.8 ± 279.6
		5	3	5	48.8 ± 8.8	11.8 ± 0.2	−92.0 ± 4.8	3005.0 ± 78.4
		6	6	4	52.5 ± 32.1	1.2 ± 0.7	−88.4 ± 10.1	3184.8 ± 180.9
		7	4	5	74.5 ± 39.0	12.4 ± 0.2	−67.0 ± 12.5	2618.4 ± 233.5
		8	4	5	64.6 ± 16.1	13.5 ± 0.1	−82.0 ± 9.0	2744.4 ± 73.5

Note: HADDOCK Score: The reported values represent the average HADDOCK score of the top-ranked cluster for each docking condition. Lower scores indicate more favorable docking. Absolute score values are used for comparative purposes across variants and conditions. Cluster # is used to indicate the cluster identifier assigned by HADDOCK.

## Data Availability

The data supporting the conclusions of this article will be made available by the authors on reasonable request.

## Supplementary Materials

The following supporting information can be downloaded at: https://www.mdpi.com/article/10.3390/ijms27021054/s1.
