# Supplementary material for: In Silico Design and Characterization of a Rationally Engineered Cas12j2 Gene Editing System for the Treatment of HPV-Associated Cancers
Source: Int J Mol Sci. 2026 Jan 21;27(2):1054. doi: 10.3390/ijms27021054 (PMC12841931; doi:10.3390/ijms27021054)
Supplement: Supplementary file 1 [file ijms-27-01054-s001.zip › ijms-4064134-supplementary.pdf]

Supplementary Data

ColabFold Supplementary Data

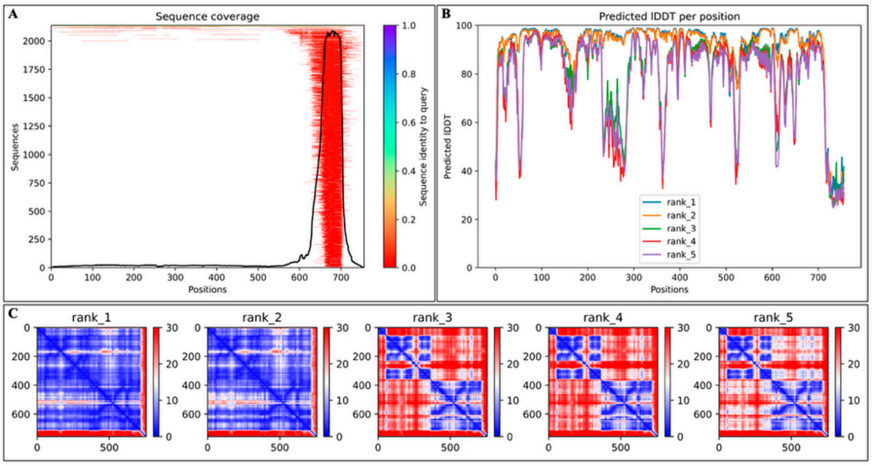

Figure S1. ColabFold Confidence Metrics for Cas12j2_WT. (A) The line graph shows the coverage plot for the multiple sequence alignment of Cas12j2_WT. (B) A line graph of per-residue predicted local distance difference test (pLDDT) scores for the five models predicted by AlphaFold2. (C) The predicted error alignment (PAE) for the five models predicted by AlphaFold2 is shown here.

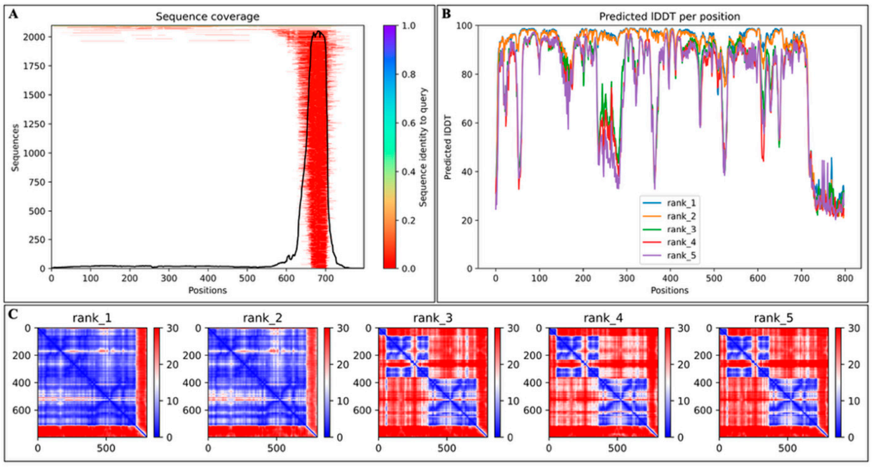

Figure S2. ColabFold Confidence Metrics for Cas12j2_F1. (A) The line graph shows the coverage plot for the multiple sequence alignment of Cas12j2_F1. (B) Line graph of per-residue predicted local distance difference test (pLDDT) scores for the five models predicted by AlphaFold2. (C) The predicted error alignment (PAE) for the five models predicted by AlphaFold2 is shown here.

RMSD Figures

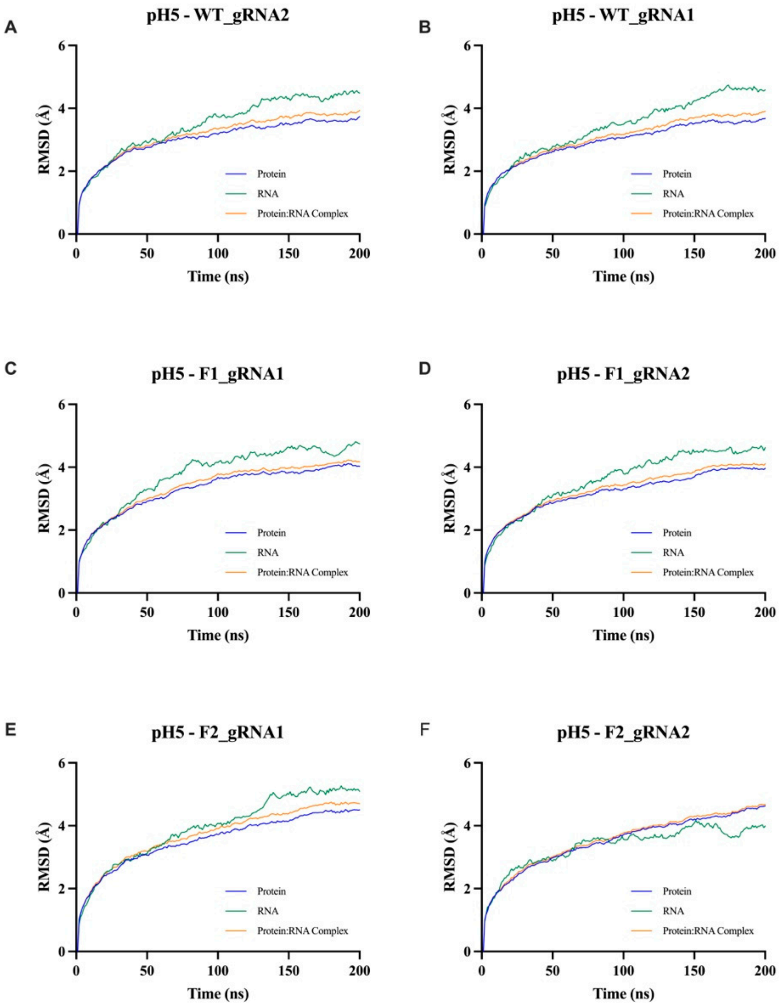

Figure S3. Root Mean Squared Deviation (RMSD) of Cas12j2-gRNA Complexes at pH 5. (A) WT_gRNA1 RMSD at pH 5. (B) WT_gRNA2 RMSD at pH 5. (C) F1_gRNA1 RMSD at pH 5. (D) F1_gRNA2 RMSD at pH 5. (E) F2_gRNA1 RMSD at pH 5. (F) F2_gRNA2 RMSD at pH 5.

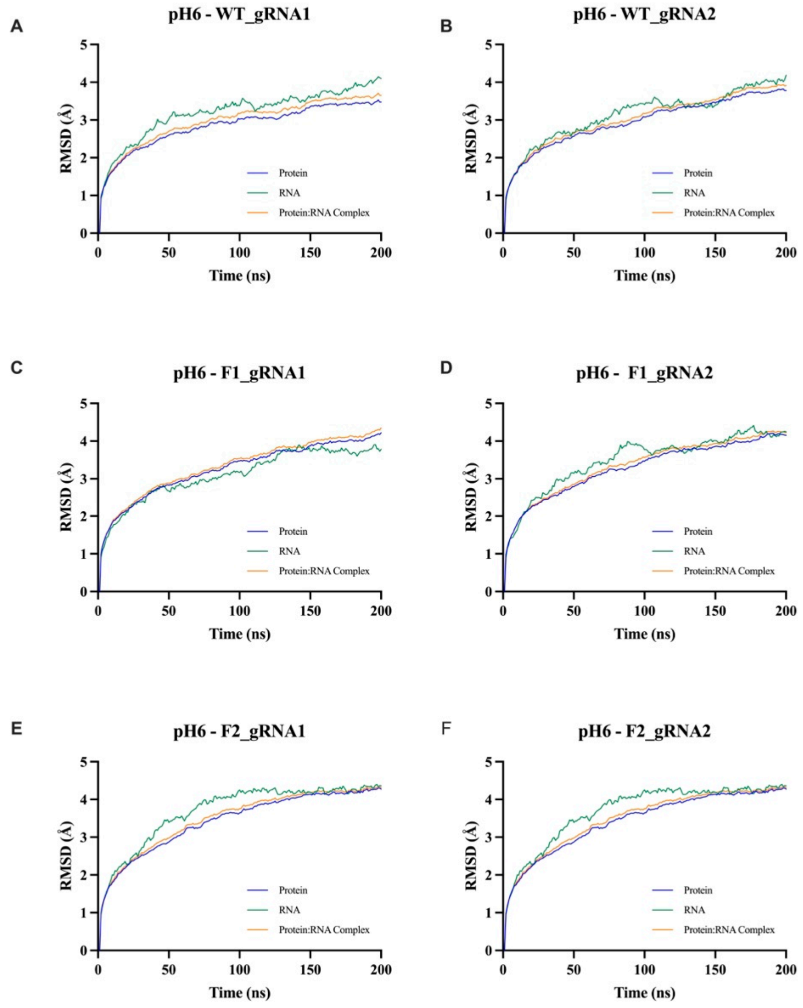

Figure S4. Root Mean Squared Deviation (RMSD) of Cas12j2-gRNA Complexes at pH 6. (A) WT_gRNA1 RMSD at pH 6. (B) WT_gRNA2 RMSD at pH 6. (C) F1_gRNA1 RMSD at pH 6. (D) F1_gRNA2 RMSD at pH 6. (E) F2_gRNA1 RMSD at pH 6. (F) F2_gRNA2 RMSD at pH 6.

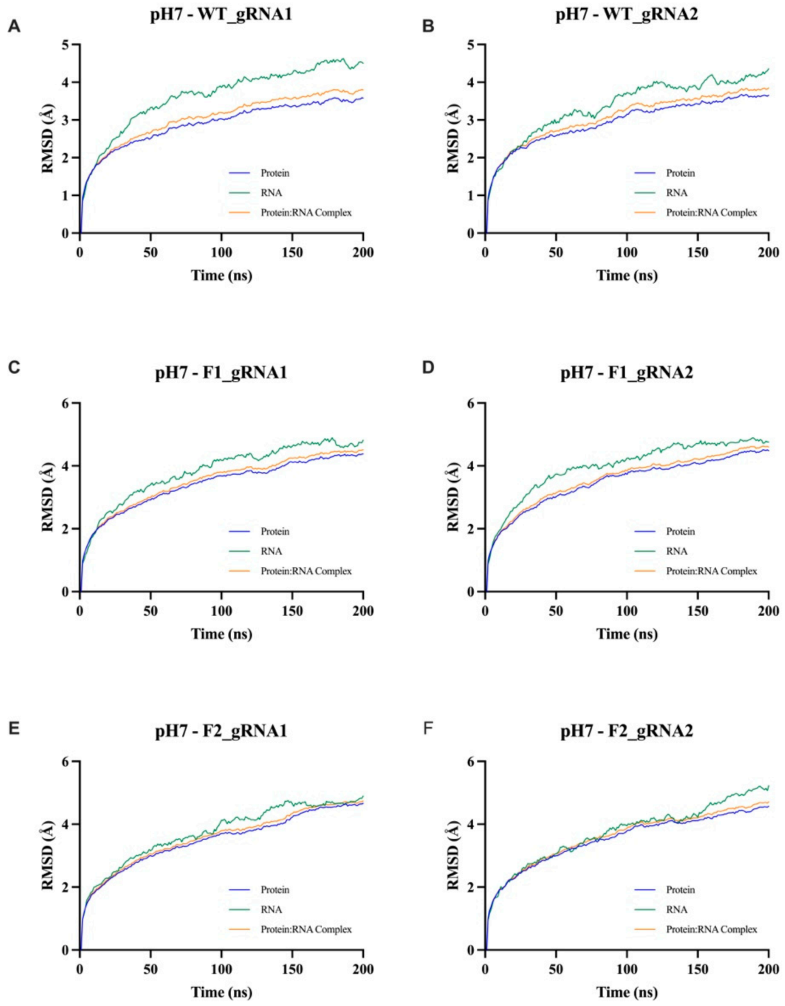

Figure S5. Root Mean Squared Deviation (RMSD) of Cas12j2-gRNA Complexes at pH 7. (A) WT_gRNA1 RMSD at pH 7. (B) WT_gRNA2 RMSD at pH 7. (C) F1_gRNA1 RMSD at pH 7. (D) F1_gRNA2 RMSD at pH 7. (E) F2_gRNA1 RMSD at pH 7. (F) F2_gRNA2 RMSD at pH 7.

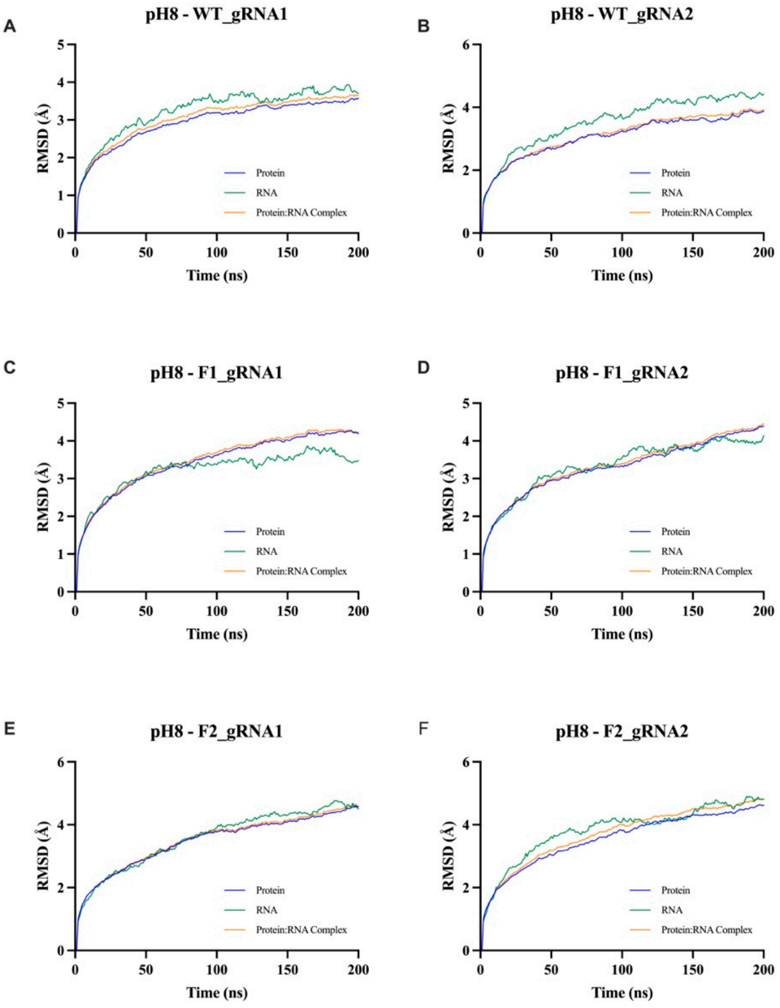

Figure S6. Root Mean Squared Deviation (RMSD) of Cas12j2-gRNA Complexes at pH 8. (A) WT_gRNA1 RMSD at pH 8. (B) WT_gRNA2 RMSD at pH 8. (C) F1_gRNA1 RMSD at pH 8. (D) F1_gRNA2 RMSD at pH 8. (E) F2_gRNA1 RMSD at pH 8. (F) F2_gRNA2 RMSD at pH 8.

RMSF Figures

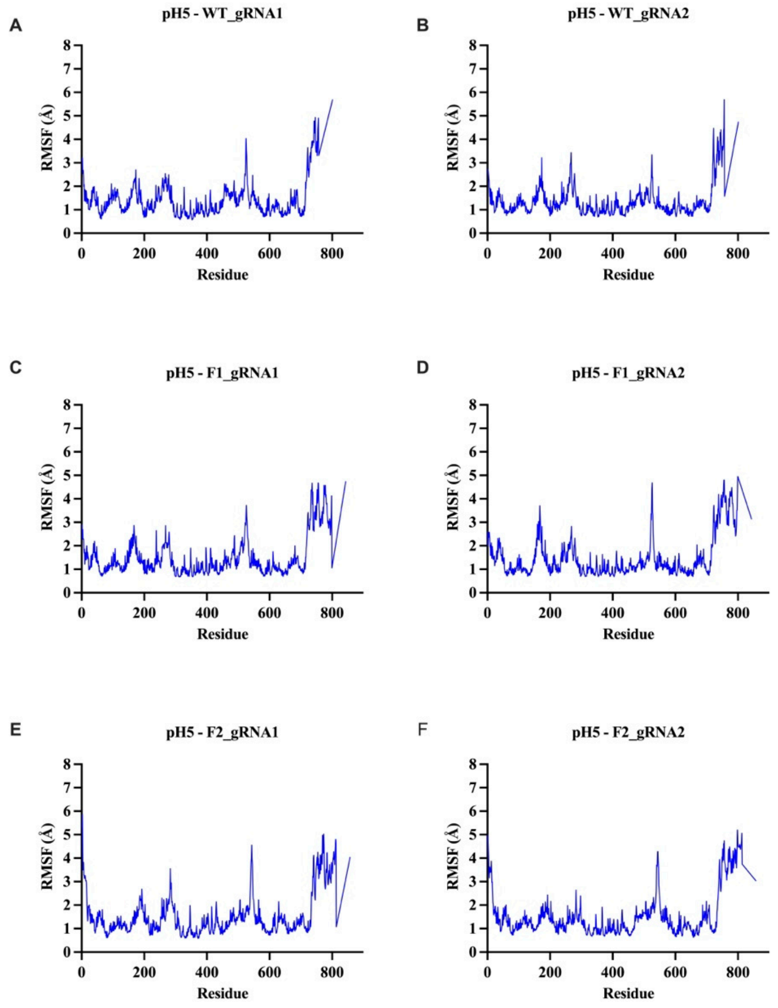

Figure S7. Root Mean Squared Fluctuation (RMSF) of Cas12j2-gRNA Complexes at pH 5. (A) WT_gRNA1 RMSF at pH 5. (B) WT_gRNA2 RMSF at pH 5. (C) F1_gRNA1 RMSF at pH 5. (D) F1_gRNA2 RMSF at pH 5. (E) F2_gRNA1 RMSF at pH 5. (F) F2_gRNA2 RMSF at pH 5.

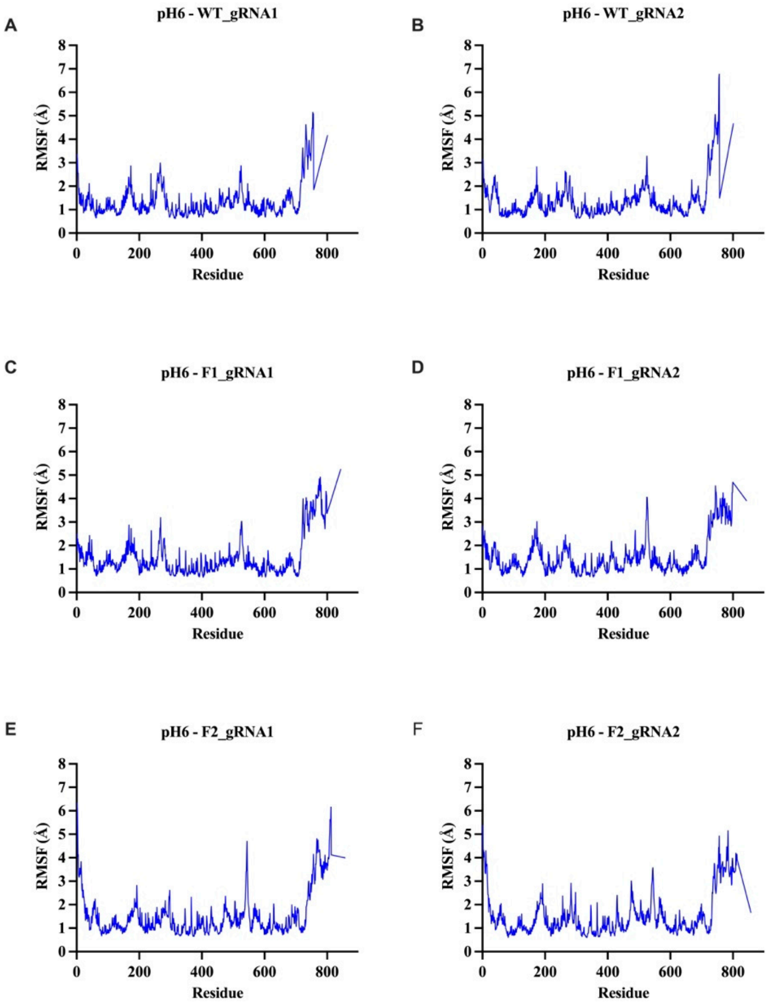

Figure S8. Root Mean Squared Fluctuation (RMSF) of Cas12j2-gRNA Complexes at pH 6. (A) WT_gRNA1 RMSF at pH 6. (B) WT_gRNA2 RMSF at pH 6. (C) F1_gRNA1 RMSF at pH 6. (D) F1_gRNA2 RMSF at pH 6. (E) F2_gRNA1 RMSF at pH 6. (F) F2_gRNA2 RMSF at pH 6.

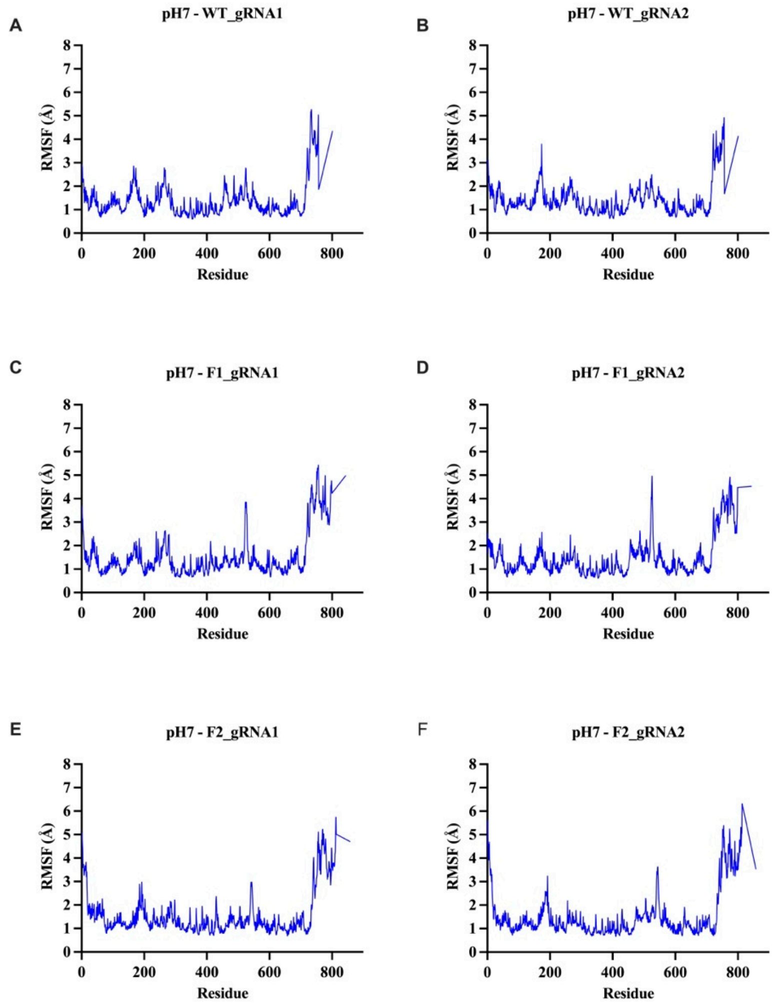

Figure S9. Root Mean Squared Fluctuation (RMSF) of Cas12j2-gRNA Complexes at pH 7. (A) WT_gRNA1 RMSF at pH 7. (B) WT_gRNA2 RMSF at pH 7. (C) F1_gRNA1 RMSF at pH 7. (D) F1_gRNA2 RMSF at pH 7. (E) F2_gRNA1 RMSF at pH 7. (F) F2_gRNA2 RMSF at pH 7.

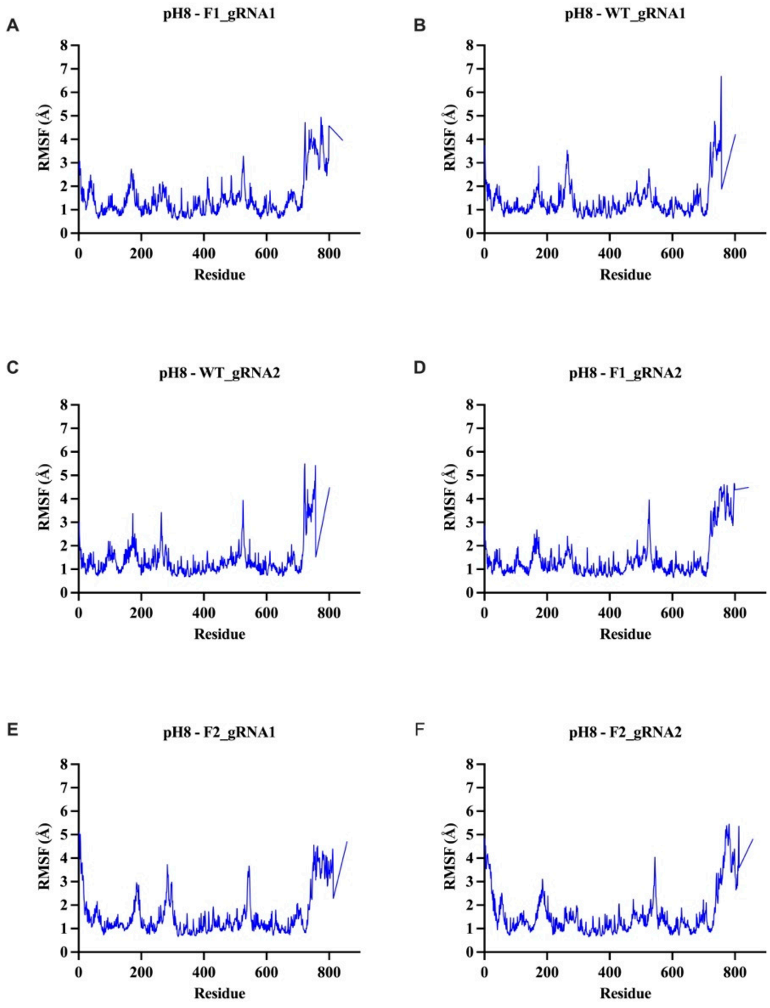

Figure S10. Root Mean Squared Fluctuation (RMSF) of Cas12j2-gRNA Complexes at pH 8. (A) WT_gRNA1 RMSF at pH 8. (B) WT_gRNA2 RMSF at pH 8. (C) F1_gRNA1 RMSF at pH 8. (D) F1_gRNA2 RMSF at pH 8. (E) F2_gRNA1 RMSF at pH 8. (F) F2_gRNA2 RMSF at pH 8.

Rg Figures

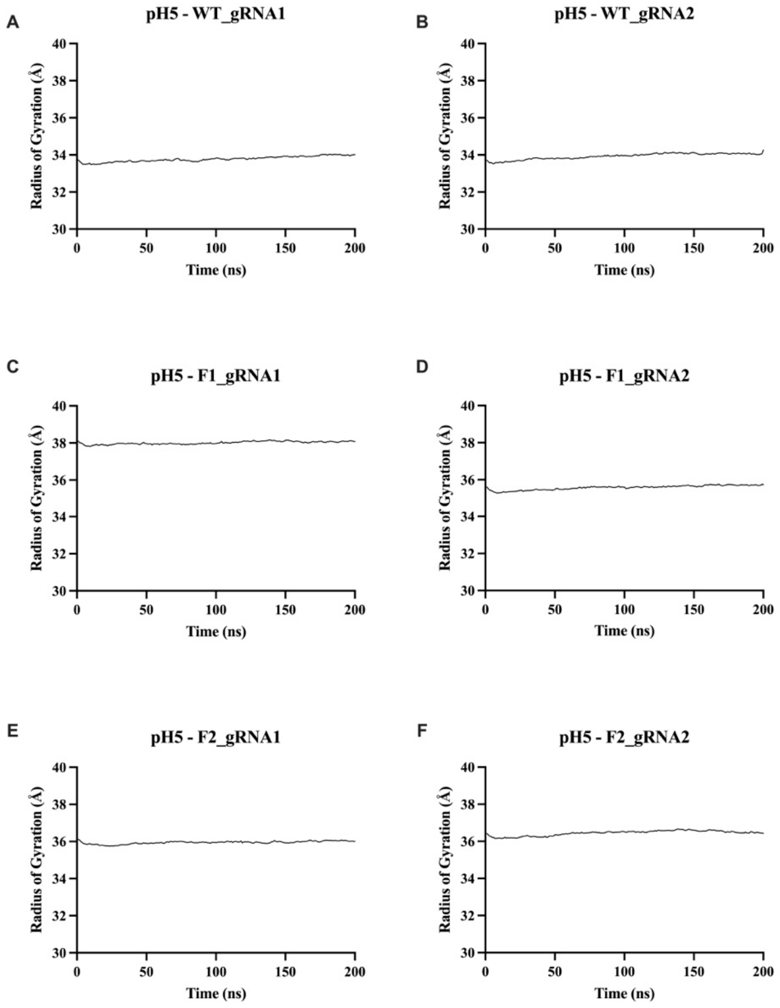

Figure S11. Radius of Gyration (Rg) of Protein-RNA Complexes at pH 5. (A) WT_gRNA1 Rg at pH 5. (B) WT_gRNA2 Rg at pH 5. (C) F1_gRNA1 Rg at pH 5. (D) F1_gRNA2 Rg at pH 5. (E) F2_gRNA1 Rg at pH 5. (F) F2_gRNA2 Rg at pH 5.

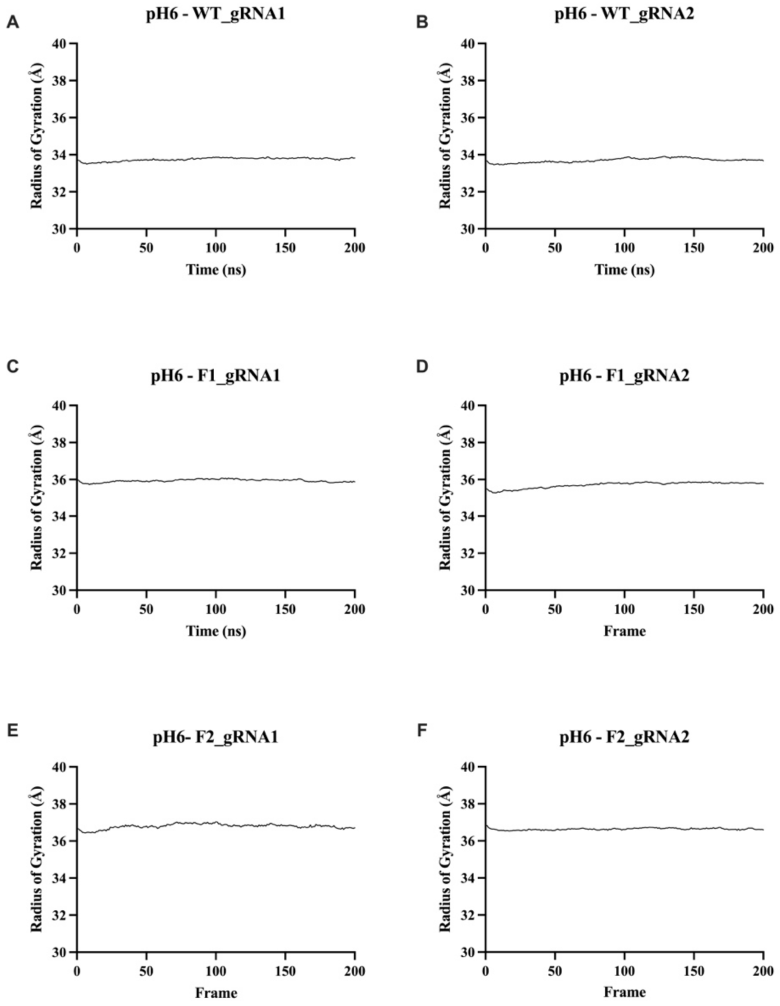

Figure S12. Radius of Gyration (Rg) of Protein-RNA Complexes at pH 6. (A) WT_gRNA1 Rg at pH 6. (B) WT_gRNA2 Rg at pH 6. (C) F1_gRNA1 Rg at pH 6. (D) F1_gRNA2 Rg at pH 6. (E) F2_gRNA1 Rg at pH 6. (F) F2_gRNA2 Rg at pH 6.

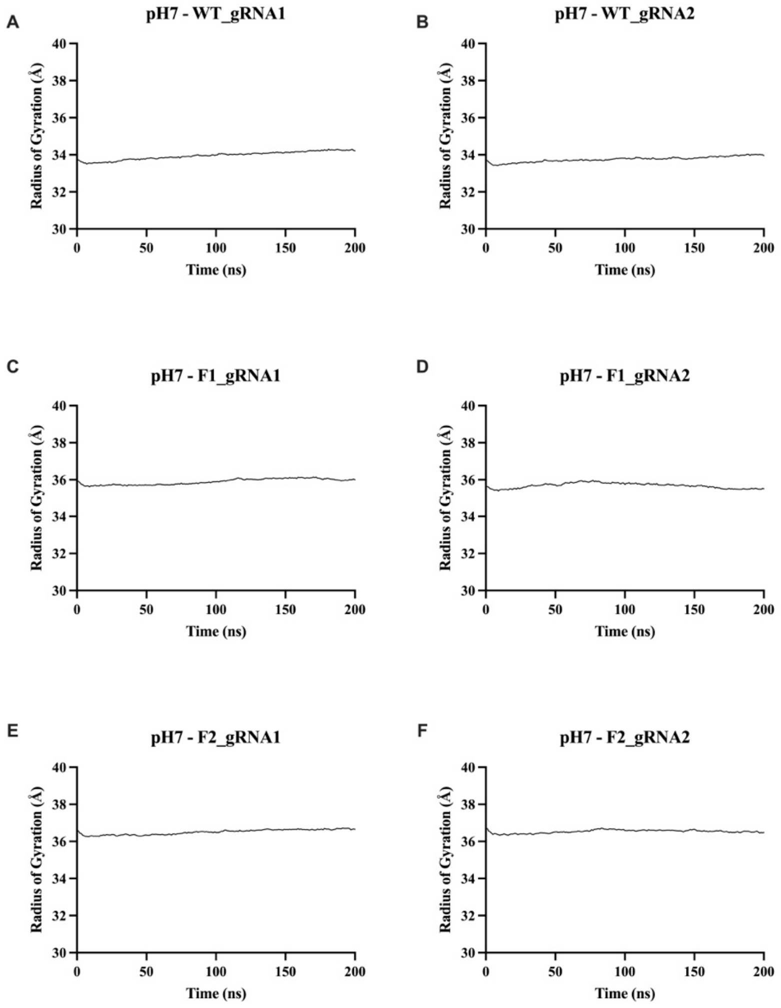

Figure S13. Radius of Gyration (Rg) of Protein-RNA Complexes at pH 7. (A) WT_gRNA1 Rg at pH 7. (B) WT_gRNA2 Rg at pH 7. (C) F1_gRNA1 Rg at pH 7. (D) F1_gRNA2 Rg at pH 7. (E) F2_gRNA1 Rg at pH 7. (F) F2_gRNA2 Rg at pH 7.

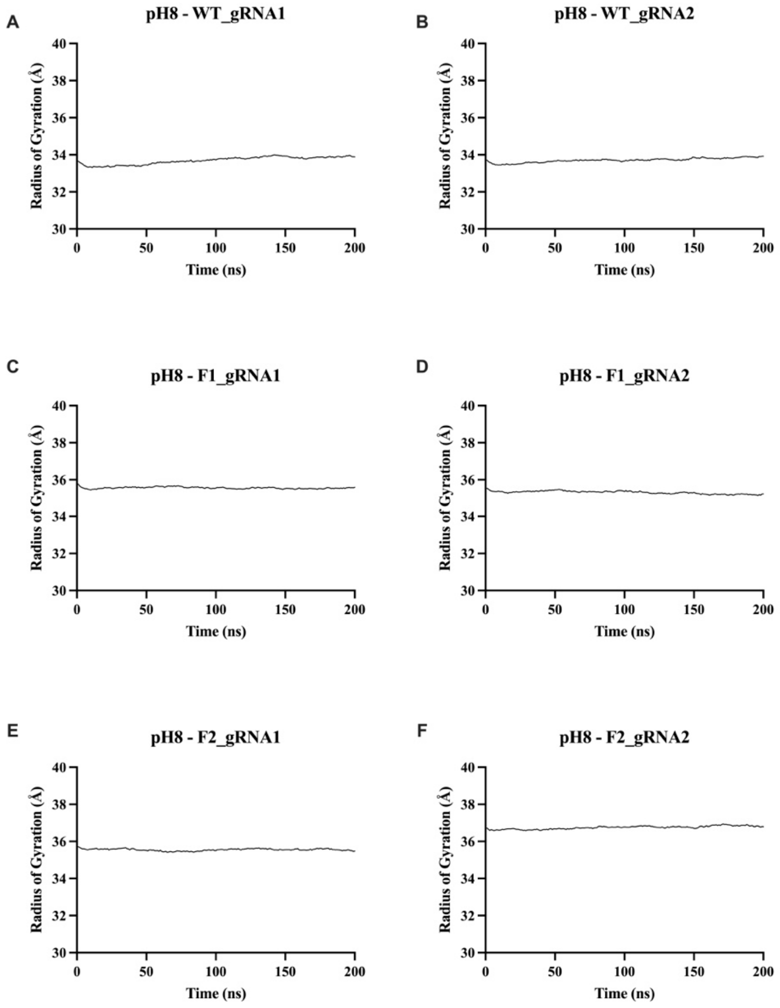

Figure S14. Radius of Gyration (Rg) of Protein-RNA Complexes at pH 8. (A) WT_gRNA1 Rg at pH 8. (B) WT_gRNA2 Rg at pH 8. (C) F1_gRNA1 Rg at pH 8. (D) F1_gRNA2 Rg at pH 8. (E) F2_gRNA1 Rg at pH 8. (F) F2_gRNA2 Rg at pH 8.

Electrostatic Maps Figures

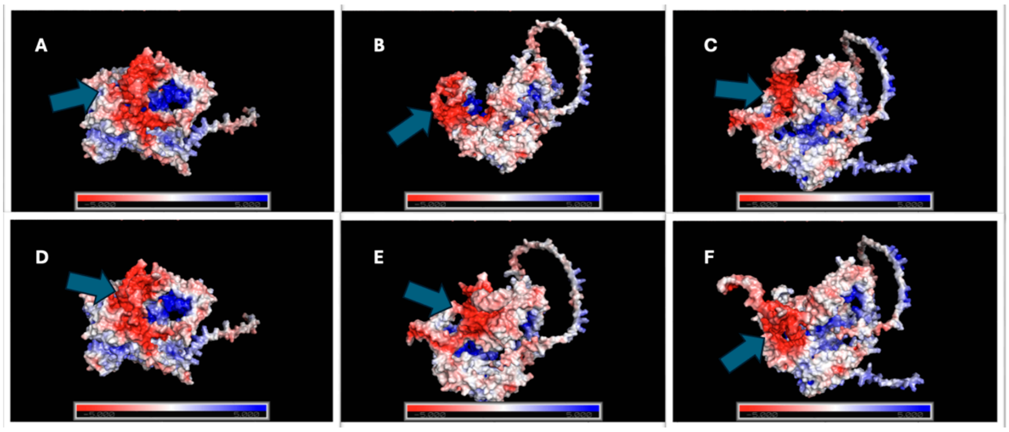

Figure S15. Electrostatic Surface Maps of Cas12j2 Fusion Constructs Bound to gRNAs at pH 5. **(A–C)** Cas12j2 fusion constructs bound to gRNA1. (A) Cas12j2_WT-gRNA1. (B) Cas12j2_F1-gRNA1. (C) Cas12j2_F2-gRNA1. (D-F) Cas12j2 fusion constructs bound to gRNA2. (D) Cas12j2_WT-gRNA2. (E) Cas12j2_F1-gRNA2. (F) Cas12j2_F2-gRNA2.

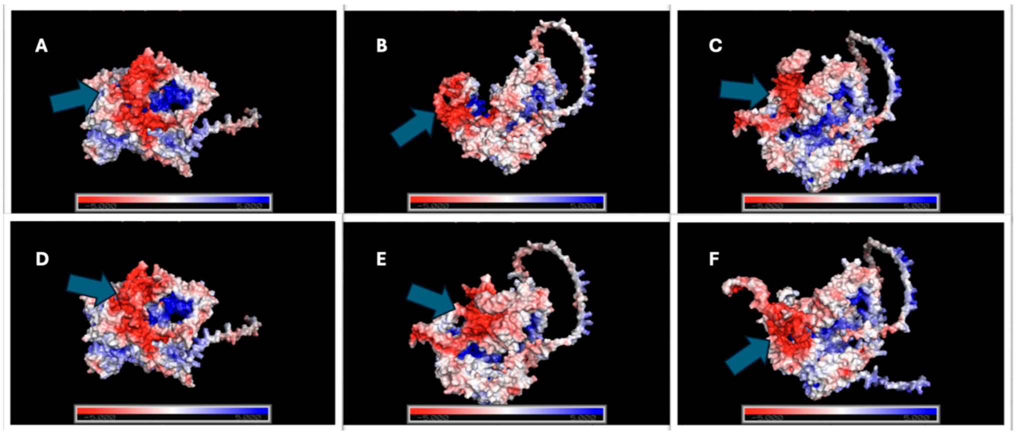

Figure S16. Electrostatic Surface Maps of Cas12j2 Fusion Constructs Bound to gRNAs at pH 6. **(A–C)** Cas12j2 fusion constructs bound to gRNA1. (A) Cas12j2_WT-gRNA1. (B) Cas12j2_F1-gRNA1. (C) Cas12j2_F2-gRNA1. (D-F) Cas12j2 fusion constructs bound to gRNA2. (D) Cas12j2_WT-gRNA2. (E) Cas12j2_F1-gRNA2. (F) Cas12j2_F2-gRNA2.

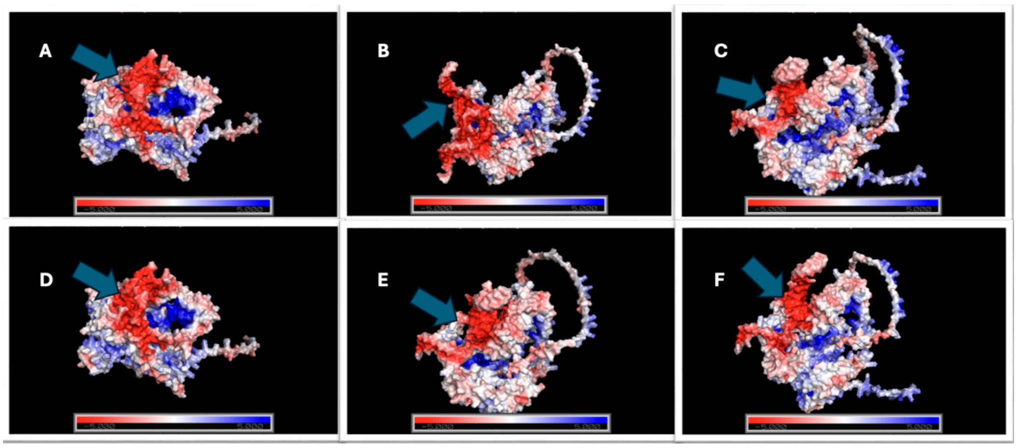

Figure S17. Electrostatic Surface Maps of Cas12j2 Fusion Constructs Bound to gRNAs at pH 7. **(A–C)** Cas12j2 fusion constructs bound to gRNA1. (A) Cas12j2_WT-gRNA1. (B) Cas12j2_F1-gRNA1. (C) Cas12j2_F2-gRNA1. (D-F) Cas12j2 fusion constructs bound to gRNA2. (D) Cas12j2_WT-gRNA2. (E) Cas12j2_F1-gRNA2. (F) Cas12j2_F2-gRNA2.

Supplemental Table S1. Raw Performance Metrics for Cas12j2:gRNA pH-Dependent Simulations in HADDOCK 2.4.

Variant ID	gRNA	pH	Cluster #	Cluster Size	HADDOCK Score (±SD)	RMSD (±SD)	vdW Energy (kcal/mol±SD)	Electrostatic Energy (kcal/mol±SD)	Desolvation Energy (kcal/mol±SD)	Restraint Violation Energy (kcal/mol±SD)	BSA (Å^2±SD)	Z-score
Cas12j2_WT	1	4	2	22	33.0±16.6	16.3±0.1	-85.0±8.8	-679.9±57.8	39.6 ±3.7	2144.1±124.6	3093.2±134.4	-1.8
		5	4	6	30.1±38.6	0.8±0.5	-102.0±13.9	-526.1±56.7	36.1±4.5	2012.2±201.9	3293.0±188.1	-1.7
		6	2	19	32.6±16.2	16.3±0.1	-81.2±3.8	-685.0±53.2	39.2±3.8	2116.4±84.5	3035.6±108.0	-1.8
		7	2	17	43.6±7.0	16.3±0.1	-85.2±8.7	-646.5±15.9	37.7±1.4	2203.5±87.6	3039.6±114.8	-1.6
		8	2	19	32.6±16.2	16.3±0.1	-81.2±3.8	-685.0±53.2	39.2±3.8	2116.4±84.5	3035.6±108.0	-1.7
	2	4	2	16	46.3±11.2	6.4±0.3	-79.2±11.3	-550.0±26.9	32.1±6.1	2034.4±88.2	2912.0±249.9	-2
		5	2	11	46.5±11.4	6.5±0.3	-80.2±11.6	-558.5±16.7	32.9±5.1	2054.6±121.2	2935.2±248.1	-2.2
		6	2	17	22.4±8.2	6.3±0.1	-97.8±16.7	-623.9±47.4	38.5±1.6	2065.3±128.6	3455.1±306.4	-1.9
		7	2	11	26.2±9.2	6.3±0.1	-92.9±9.5	-574.4±26.9	37.8±2.2	1962.3±29.1	3223.8±143.7	-2.1
		8	2	17	32.1±11.1	6.2±0.0	-86.5±3.4	-580.2±28.1	34.3±3.9	2002.4±79.1	3140.2±131.9	-2.3
Cas12j2_F1	1	4	2	11	108.1±23.9	12.5±0.6	-62.2±14.2	-480.0±51.8	26.2±2.8	2401.4±119.0	2199.6±230.7	-1.6
		5	1	19	109.0±11.1	13.0±0.0	-64.7±8.2	-436.4±82.2	21.5±3.6	2395.6±112.0	2168.7±302.3	-2.1
		6	3	6	99.6±5.1	12.6±0.3	-93.5±9.1	-436.9±43.7	28.6±3.0	2518.2±76.8	2773.5±235.9	-1.7
		7	5	6	95.4±35.7	10.9±0.5	-73.4±14.4	-533.5±65.9	28.5±11.3	2471.1±240.1	2638.0±369.1	-1.3
		8	8	4	132.4±27.9	10.7±0.2	-60.3±3.9	-500.6±49.7	25.1±8.9	2676.4±271.6	2307.6±135.7	-1.4
	2	4	3	6	82.8±22.2	14.0±0.1	-89.5±10.3	-545.2±94.2	46.7±3.7	2346.1±88.6	2876.9±140.8	-2.3
		5	5	6	96.2±13.0	4.6±0.1	-96.5±4.5	-453.1±31.4	40.3±3.9	2429.4±95.5	3260.3±263.8	-1.3

		6	5	8	91.8±14.8	8.6±0.4	-80.4±11.9	-520.8±79.4	42.5±7.9	2337.8±98.4	2647.2±313.9	-1.4
		7	5	6	103.8±29.8	7.9±0.1	-70.2±18.6	-530.6±80.7	40.9±7.8	2391.9±94.2	2614.7±314.2	-1.7
		8	3	7	103.6±20.3	12.8±0.1	-85.7±8.8	-493.2±47.7	49.2±1.3	2386.9±107.0	2680.9±204.7	-1.7
Cas12j2_F2	1	4	2	12	40.7±11.5	17.1±0.3	-94.5±3.2	-544.7±67.3	32.8±7.0	2113.2±105.5	3079.6±262.1	-1.6
		5	1	15	109.4±12.4	11.5±0.4	-83.3±6.8	-313.4±84.1	37.5±8.8	2179.6±34.9	2632.5±258.8	-1.2
		6	2	7	51.4±5.0	13.6±0.2	-90.7±2.6	-541.7±25.9	40.9±2.1	2094.3±93.4	2890.7±102.7	-1.7
		7	3	5	70.7±19.7	12.9±0.7	-80.4±18.4	-562.1±42.8	43.4±3.8	2201.7±130.0	2854.2±243.1	-1.3
		8	3	8	66.6±15.3	16.1±0.1	-86.1±5.9	-515.5±114.6	35.0±8.1	2208.2±121.2	2935.4±235.7	-1.2
	2	4	5	4	79.5±21.0	12.6±0.1	-77.2±9.8	-494.8±97.8	41.9±4.7	2137.9±91.0	2695.8±279.6	-1.9
		5	3	5	48.8±8.8	11.8±0.2	-92.0±4.8	-527.0±47.9	51.5±4.3	1947.3±131.8	3005.0±78.4	-1.7
		6	6	4	52.5±32.1	1.2±0.7	-88.4±10.1	-585.6±66.6	35.2±5.7	2228.1±195.8	3184.8±180.9	-1.4
		7	4	5	74.5±39.0	12.4±0.2	-67.0±12.5	-550.6±81.2	42.3±4.9	2092.7±185.0	2618.4±233.5	-1.5
		8	4	5	64.6±16.1	13.5±0.1	-82.0±9.0	-587.5±60.9	43.5±4.1	2206.7±52.6	2744.4±73.5	-1.4

Note: **HADDOCK Score**: The reported values represent the average HADDOCK score of the top-ranked cluster for each docking condition. Lower scores indicate more favorable docking. Absolute score values are used for comparative purposes across variants and conditions.

Supplemental Table S2. Cas12j2 Variant Sequences:

Cas12j2_WT	atgccaaagccagccgtggagtctgagttttctaaggtactcaagaagcactttccgggcgagcg atttaggtctagctacatgaagcggggtggtaaaatcttggcagcccagggtgaagaagcggtc gtcgcgtatctgcaaggcaagtccgaggaggaacccccgaattttcagccgccggcgaaatgtc atgttgttacgaaatcacgagatttcgccgagtggccaattatgaaggcctccgaagcaatccaa aggtatatctatgcgctctctacgacggaacgggcagcttgcaagcctggcaaatcttcagagtc

	ccacgcggcctggttcgcggcaactggcgtgtcaaaccacggttatagccatgttcaaggcctca<br>atcttatcttcgaccacacgctgggaagatacgatggtgttctgaaaaaggtgcagctgagaaat<br>gagaaagcccgcgcccggctggaaagtatcaacgcctctcgagccgacgaaggacttccagaa<br>ataaaggcagaggaggaagaggtcgctacaaatgaaaccggacaccttttgcagcctccggg<br>gatcaacccaagtttctacgtttaccagactatttctccgcaggcttacaggccgcgagatgagatt<br>gtactgccgcccgagtatgccggctacgtccgagatccgaacgcccctatccccсttggcgtggtt<br>cggaatcggtgcgatattcagaagggatgccctggatacatccccgaatggcaaagagaggca<br>ggtactgcaatttcccctaagacgggtaaagccgtcaccgttcccggcctcagtccaaaaaaaaa<br>taaacgaatgcgacgatactggaggtccgagaaagagaaggcccaagatgcactgctcgttac<br>tgtgagaatcggcactgactgggtcgtaatcgacgttcgaggtttgctgcggaatgcgcggtgg<br>cgcaccattgcgcccaaggatatatccttgaatgccctcttggatctctttacaggcgacccggtca<br>tagatgttcggagaaacattgtgactttcacctacactctggacgcttgcggtacatatgctcgcaa<br>atggactctcaaagggaaacagactaaggcaaccctcgataagttgaccgcaacccagaccgt<br>ggccctggtagcaatagaccttggacaaaccaatcccataagtgcgggtatcagtagggtcacg<br>caagaaaacggggcacttcaatgtgaacctctggatcggttcactctccctgatgatctgctcaag<br>gatatctccgcgtaccgaatcgcttgggatcgcaacgaggaggaactgagggctaggtccgtc<br>gaagcgctcccagaagctcaacaagctgaagtgagggctctggacggcgtttctaaagaaacc<br>gccaggacccagctctgcgcggacttcggccttgatcccaaacggctgccttgggataaaatga<br>gcagcaacaccactttcatcagtgaagcgttgcttagtaattctgtgtctagagatcaggtttttta<br>ctcctgcgcctaaaaagggagcaaagaaaaaagcccccgttgaagttatgcggaaggatagga<br>cctgggcgagggcctataaaccacggctcagtgtggaagcccaaaagctgaaaaatgaggcct<br>tgtgggctctcaagcgcacttctccagaatacctcaagctgagtcggagaaaagaggagctttgt<br>aggcgaagtattaactacgtcattgaaaaaacaagacggaggacacaatgtcagatcgtgatac<br>ctgtcatagaggacttgaatgtgcgattctttcacggttcagggaagcgcctgcctggctgggata<br>atttttcactgcgaagaaggagaacaggtggtttatacagggcctccacaaagcattcagcgact<br>tgcgaactcatcgctccttctacgtattcgaagtccgcccggagcggacttcaataacgtgcccaa<br>aatgcgggcactgcgaggttgggaaccgggatggggaggcttttcagtgccttagttgcggcaa<br>aacgtgcaatgccgaccttgacgtggctacccataatctgactcaagtcgcccttacaggaaaaa

	caatgccgaaacgcgaggaacctagagatgcccagggcacagctccagcccgaaaaacaaag<br>aaggcgtcaaagagcaaggctccgccagccgaacgagaggaccaaactccagcacaggaac<br>cgtcccagacttcc
Cas12j2_F1	atgccaaagccagccgtggagtctgagttttctaaggtactcaagaagcactttccgggcgagcg<br>atttaggtctagctacatgaagcggggtggtaaaatcttggcagcccagggtgaagaagcggtc<br>gtcgcgtatctgcaaggcaagtccgaggaggaacccccgaattttcagccgccggcgaaatgtc<br>atgttgttacgaaatcacgagatttcgccgagtggccaattatgaaggcctccgaagcaatccaa<br>aggtatatctatgcgctctctacgacggaacgggcagcttgcaagcctggcaaatcttcagagtc<br>ccacgcggcctggttcgcggcaactggcgtgtcaaaccacggttatagccatgttcaaggcctca<br>atcttatcttcgaccacacgctgggaagatacgatggtgttctgaaaaaggtgcagctgagaaat<br>gagaaagcccgcgcccggctggaaagtatcaacgcctctcgagccgacgaaggacttccagaa<br>ataaaggcagaggaggaagaggtcgctacaaatgaaaccggacaccttttgcagcctccggg<br>gatcaacccaagtttctacgtttaccagactatttctccgcaggcttacaggccgcgagatgagatt<br>gtactgccgcccgagtatgccggctacgtccgagatccgaacgcccctatcccccttggcgtggtt<br>cggaatcggtgcgatattcagaagggatgccctggatacatccccgaatggcaaagagaggca<br>ggtactgcaatttcccctaagacgggtaaagccgtcaccgttcccggcctcagtccaaaaaaaaa<br>taaacgaatgcgacgatactggaggtccgagaaagagaaggcccaagatgcactgctcgttac<br>tgtgagaatcggcactgactgggtcgtaatcgacgttcgaggtttgctgcggaatgcgcggtgg<br>cgcaccattgcgcccaaggatatatccttgaatgccctcttggatctctttacaggcgacccggtca<br>tagatgttcggagaaacattgtgactttcacctacactctggacgcttgcggtacatatgctcgcaa<br>atggactctcaaagggaaacagactaaggcaaccctcgataagttgaccgcaacccagaccgt<br>ggccctggtagcaatagaccttggacaaaccaatcccataagtgcgggtatcagtagggtcacg<br>caagaaaacggggcacttcaatgtgaacctctggatcggttcactctccctgatgatctgctcaag<br>gatatctccgcgtaccgaatcgcttgggatcgcaacgaggaggaactgagggctaggtccgtc<br>gaagcgctcccagaagctcaacaagctgaagtgagggctctggacggcgtttctaaagaaacc<br>gccaggacccagctctgcgcggacttcggccttgatcccaaacggctgccttgggataaaatga<br>gcagcaacaccactttcatcagtgaagcgttgcttagtaattctgtgtctagagatcaggttttttta

	ctcctgcgcctaaaaagggagcaaagaaaaaagcccccgttgaagttatgcggaaggatagga<br>cctgggcgagggcctataaaccacggctcagtgtggaagcccaaaagctgaaaaatgaggcct<br>tgtgggctctcaagcgcacttctccagaatacctcaagctgagtcggagaaaagaggagctttgt<br>aggcgaagtattaactacgtcattgaaaaaacaagacggaggacacaatgtcagatcgtgatac<br>ctgtcatagaggacttgaatgtgcgattctttcacggttcagggaagcgcctgcctggctgggata<br>attttttcactgcgaagaaggagaacaggtggtttatacagggcctccacaaagcattcagcgact<br>tgcgaactcatcgctccttctacgtattcgaagtccgcccggagcggacttcaataacgtgcccaa<br>aatgcgggcactgcgaggttgggaaccgggatggggaggcttttcagtgccttagttgcggcaa<br>aacgtgcaatgccgaccttgacgtggctacccataatctgactcaagtcgcccttacaggaaaaa<br>caatgccgaaacgcgaggaacctagagatgcccagggcacagctccagcccgaaaaacaaag<br>aaggcgtcaaagagcaaggctccgccagccgaacgagaggaccaaactccagcacaggaac<br>cgtcccagacttccggaagcggacccaagaaaaaacgcaaggtggaagatcctaagaaaaag<br>cggaaagtgagcctgggcagcggctccgattacaaagatgacgatgacaaagactacaagga<br>tgatgatgataag
Cas12j2_F2	cccaagaaaaaacgcaaggtgggaggacctaagaaaaagcggaaagtgggaagcggacca<br>aagccagccgtggagtctgagttttctaaggtactcaagaagcactttccgggcgagcgatttag<br>gtctagctacatgaagcggggtggtaaaatcttggcagcccagggtgaagaagcggtcgtcgc<br>gtatctgcaaggcaagtccgaggaggaacccccgaattttcagccgccggcgaaatgtcatgtt<br>gttacgaaatcacgagatttcgccgagtggccaattatgaaggcctccgaagcaatccaaaggta<br>tatctatgcgctctctacgacggaacgggcagcttgcaagcctggcaaatcttcagagtcccacg<br>cggcctggttcgcggcaactggcgtgtcaaaccacggttatagccatgttcaaggcctcaatctta<br>tcttcgaccacacgctgggaagatacgatggtgttctgaaaaaggtgcagctgagaaatgagaa<br>agcccgcgcccggctggaaagtatcaacgcctctcgagccgacgaaggacttccagaaataaa<br>ggcagaggaggaagaggtcgctacaaatgaaaccggacaccttttgcagcctccggggatcaa<br>cccaagtttctacgtttaccagactatttctccgcaggcttacaggccgcgagatgagattgtactg<br>ccgcccgagtatgccggctacgtccgagatccgaacgcccctatccccccttggcgtggttcggaa<br>tcggtgcgatattcagaagggatgccctggatacatccccgaatggcaaagagaggcaggtac

	tgcaatttcccctaagacgggtaaagccgtcaccgttcccggcctcagtccaaaaaaaataaac<br>gaatgcgacgatactggaggtccgagaaagagaaggcccaagatgcactgctcgttactgtga<br>gaatcggcactgactgggtcgtaatcgacgttcgaggtttgctgcggaatgcgcggtggcgcac<br>cattgcgcccaaggatatatccttgaatgccctcttggatctctttacaggcgacccggtcatagat<br>gttcggagaaacattgtgactttcacctacactctggacgcttgcggtacatatgctcgcaaatgg<br>actctcaaagggaaacagactaaggcaaccctcgataagttgaccgcaacccagaccgtggccc<br>tggtagcaatagaccttggacaaaccaatcccataagtgcgggtatcagtagggtcacgcaaga<br>aaacggggcacttcaatgtgaacctctggatcggttcactctccctgatgatctgctcaaggatatc<br>tccgcgtaccgaatcgcttgggatcgcaacgaggaggaactgagggctaggtccgtcgaagcg<br>ctcccagaagctcaacaagctgaagtgagggctctggacggcgtttctaaagaaaccgccagg<br>acccagctctgcgcggacttcggccttgatcccaaacggctgccttgggataaaatgagcagcaa<br>caccactttcatcagtgaagcgttgcttagtaattctgtgtctagagatcaggttttttttactcctgcg<br>cctaaaaagggagcaaagaaaaaagcccccgttgaagttatgcggaaggataggacctgggc<br>gagggcctataaaccacggctcagtgtggaagcccaaaagctgaaaaatgaggccttgtgggc<br>tctcaagcgcacttctccagaatacctcaagctgagtcggagaaaagaggagctttgtaggcga<br>agtattaactacgtcattgaaaaaacaagacggaggacacaatgtcagatcgtgatacctgtcat<br>agaggacttgaatgtgcgattctttcacggttcagggaagcgcctgcctggctgggataatttttc<br>actgcgaagaaggagaacaggtggtttatacagggcctccacaaagcattcagcgacttgcga<br>actcatcgctccttctacgtattcgaagtccgcccggagcggacttcaataacgtgcccaaaatgc<br>gggcactgcgaggttgggaaccgggatggggaggcttttcagtgccttagttgcggcaaaacgt<br>gcaatgccgaccttgacgtggctacccataatctgactcaagtcgcccttacaggaaaaacaatg<br>ccgaaacgcgaggaacctagagatgcccagggcacagctccagcccgaaaaacaaagaagg<br>cgtcaaagagcaaggctccgccagccgaacgagaggaccaaactccagcacaggaaccgtcc<br>cagacttccggaagcggacccaagaaaaaacgcaaggtgggaggacctaagaaaaagcgga<br>aagtgggaggagattacaaagatgacgatgacaaagactacaaggatgatgatgataag

Supplemental Table S3. Plasmid Component Sequences:

CBA Promoter	acattgattattgactagttattaatagtaatcaattacggggtcattagttcatagcccatatatggag<br>ttccgcgttacataacttacggtaaatggcccgcctggctgaccgcccaacgacccccgcccattgac<br>gtcaataatgacgtatgttcccatagtaacgccaatagggactttccattgacgtcaatgggtggagt<br>atttacggtaaactgcccacttggcagtacatcaagtgtatcatatgccaagtacgccccctattgacg<br>tcaatgacggtaaatggcccgcctggcattatgcccagtacatgaccttatgggactttcctacttggc<br>agtacatctacgtattagtcatcgctattaccatggtcgaggtgagccccacgttctgcttcactctccc<br>catctcccccccctccccacccccaattttgtatttatttattttttaattattttgtgcagcgatgggggcg<br>ggggggggggggggggcgcgccaggcggggcggggcggggcgaggggcggggcgggg<br>cgaggcggagaggtgcggcggcagccaatcagagcggcgcgctccgaaagtttccttttatggcg<br>aggcggcggcggcggcggccctataaaaagcgaagcgcgcggcgggcgggagtcgctgcgc<br>gctgccttcgccccgtgccccgctccgccgccgcctcgcgccgcccgccccggctctgactgaccgc<br>gttactcccacaggtgagcgggcgggacggcccttctcctccgggctgtaattagctgagcaagag<br>gtaagggtttaagggatggttggttggtggggtattaatgtttaattacctggagcacctgcctgaaa<br>tcactttttttcag
N-Terminal Dual NLS	cccaagaaaaaacgcaaggtgggaggacctaagaaaaagcggaaagtg
mCherry Flurophore	atggtgagcaagggcgaggaggataacatggccatcatcaaggagttcatgcgcttcaaggtgca<br>catggagggctccgtgaacggccacgagttcgagatcgagggcgagggcgagggccgcccta<br>cgagggcacccagaccgccaagctgaaggtgaccaagggtggccccctgcccttcgcctgggac<br>atcctgtcccctcagttcatgtacggctccaaggcctacgtgaagcaccccgccgacatccccgacta<br>cttgaagctgtccttccccgagggcttcaagtgggagcgcgtgatgaacttcgaggacggcggcgt<br>ggtgaccgtgacccaggactcctccctgcaggacggcgagttcatctacaaggtgaagctgcgcg<br>gcaccaacttcccctccgacggccccgtaatgcagaagaagaccatgggctgggaggcctcctcc<br>gagcggatgtaccccgaggacggcgccctgaagggcgagatcaagcagaggctgaagctgaa<br>ggacggcggccactacgacgctgaggtcaagaccacctacaaggccaagaagcccgtgcagctg<br>cccggcgcctacaacgtcaacatcaagttggacatcacctcccacaacgaggactacaccatcgtg<br>gaacagtacgaacgcgccgagggccgccactccaccggcggcatggacgagctgtacaagtag

Ampicillin Resistance Gene	ttaccaatgcttaatcagtgaggcacctatctcagcgatctgtctatttcgttcatccatagttgcctgac<br>tccccgtcgtgtagataactacgatacgggagggcttaccatctggccccagtgctgcaatgatacc<br>gcgagacccacgctcaccggctccagatttatcagcaataaaccagccagccggaagggccgagc<br>gcagaagtggtcctgcaactttatccgcctccatccagtctattaattgttgccgggaagctagagta<br>agtagttcgccagttaatagtttgcgcaacgttgttgccattgctacaggcatcgtggtgtcacgctcg<br>tcgtttggtatggcttcattcagctccggttcccaacgatcaaggcgagttacatgatcccccatgttgt<br>gcaaaaaagcggttagctccttcggtcctccgatcgttgtcagaagtaagttggccgcagtgttatca<br>ctcatggttatggcagcactgcataattctcttactgtcatgccatccgtaagatgcttttctgtgactgg<br>tgagtactcaaccaagtcattctgagaatagtgtatgcggcgaccgagttgctcttgcccggcgtca<br>atacgggataataccgcgccacatagcagaactttaaaagtgctcatcattggaaaacgttcttcgg<br>ggcgaaaactctcaaggatcttaccgctgttgagatccagttcgatgtaacccactcgtgcacccaac<br>tgatcttcagcatcttttactttcaccagcgtttctgggtg
C-Terminal Dual NLS and FLAG Tag	Ttaccaatgcttaatcagtgaggcacctatctcagcgatctgtctatttcgttcatccatagttgcctga<br>ctccccgtcgtgtagataactacgatacgggagggcttaccatctggccccagtgctgcaatgatac<br>cgcgagacccacgctcaccggctccagatttatcagcaataaaccagccagccggaagggccgag<br>cgcagaagtggtcctgcaactttatccgcctccatccagtctattaattgttgccgggaagctagagt<br>aagtagttcgccagttaatagtttgcgcaacgttgttgccattgctacaggcatcgtggtgtcacgctc<br>gtcgtttggtatggcttcattcagctccggttcccaacgatcaaggcgagttacatgatcccccatgtt<br>gtgcaaaaaagcggttagctccttcggtcctccgatcgttgtcagaagtaagttggccgcagtgttat<br>cactcatggttatggcagcactgcataattctcttactgtcatgccatccgtaagatgcttttctgtgact<br>ggtgagtactcaaccaagtcattctgagaatagtgtatgcggcgaccgagttgctcttgcccggcgt<br>caatacgggataataccgcgccacatagcagaactttaaaagtgctcatcattggaaaacgttcttcg<br>gggcgaaaactctcaaggatcttaccgctgttgagatccagttcgatgtaacccactcgtgcaccca<br>actgatcttcagcatcttttactttcaccagcgtttctgggtg
